# Chemically related 4,5-linked aminoglycoside antibiotics drive subunit rotation in opposite directions

**DOI:** 10.1038/ncomms8896

**Published:** 2015-07-30

**Authors:** Michael R. Wasserman, Arto Pulk, Zhou Zhou, Roger B. Altman, John C. Zinder, Keith D. Green, Sylvie Garneau-Tsodikova, Jamie H. Doudna Cate, Scott C. Blanchard

**Affiliations:** 1 Department of Physiology and Biophysics, Weill Cornell Medical College, New York, New York 10065, USA; 2 Department of Molecular and Cell Biology, University of California at Berkeley, Berkeley, California 94720, USA; 3 Department of Chemistry, University of California at Berkeley, Berkeley, California 94720, USA; 4 Tri-Institutional Training Program in Chemical Biology, Weill Cornell Medical College, Rockefeller University, Memorial Sloan-Kettering Cancer Center, New York, New York 10065, USA; 5 Department of Pharmaceutical Sciences, University of Kentucky College of Pharmacy, Lexington, Kentucky 40536, USA

## Abstract

Dynamic remodelling of intersubunit bridge B2, a conserved RNA domain of the bacterial ribosome connecting helices 44 (h44) and 69 (H69) of the small and large subunit, respectively, impacts translation by controlling intersubunit rotation. Here we show that aminoglycosides chemically related to neomycin—paromomycin, ribostamycin and neamine—each bind to sites within h44 and H69 to perturb bridge B2 and affect subunit rotation. Neomycin and paromomycin, which only differ by their ring-I 6′-polar group, drive subunit rotation in opposite directions. This suggests that their distinct actions hinge on the 6′-substituent and the drug’s net positive charge. By solving the crystal structure of the paromomycin–ribosome complex, we observe specific contacts between the apical tip of H69 and the 6′-hydroxyl on paromomycin from within the drug’s canonical h44-binding site. These results indicate that aminoglycoside actions must be framed in the context of bridge B2 and their regulation of subunit rotation.

## Abstract (web summary)

Ratchet-like rotation of the small ribosomal subunit relative to the large is essential to the translation mechanism. Here, the authors show that chemically related aminoglycoside antibiotics have distinct impacts on the nature and rate of the subunit rotation process within the intact ribosome.

## Graphical abstract

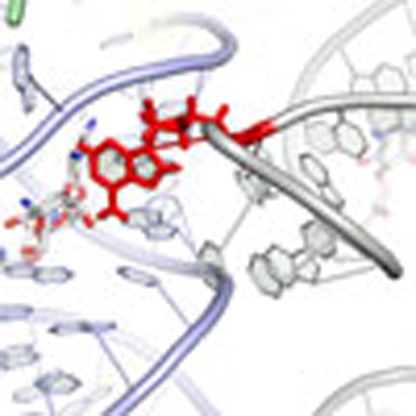

Aminoglycoside antibiotics are potent, broad-spectrum bactericidal agents that inhibit cell growth by targeting functional centres within the bacterial ribosome to alter global aspects of the translation mechanism^1^,^2^,^3^,^4^,^5^. However, despite their clinical effectiveness, aminoglycosides are commonly restricted to topical applications. Oral administration is typically a last resort to fight multi-drug-resistant pathogens due to their propensities to elicit adverse side effects in patients, including oto- and nephrotoxicities^6^. The molecular origins of aminoglycoside toxicities and their bactericidal activities are currently a matter of significant debate.

Initiatives spanning the past half century indicate that aminoglycosides with a 2-deoxystreptamine ring target the conserved helix 44 (h44) messenger RNA (mRNA) ‘decoding site’ region of ribosomal RNA (rRNA) within the small (30S) subunit of the bacterial ribosome^7^,^8^, reducing translational fidelity by promoting the promiscuous incorporation of near- and non-cognate aminoacyl-transfer RNAs (tRNAs) (miscoding)^9^,^10^. Investigations of RNA oligonucleotide model systems^2^, ribosomal subunits^11^ and intact ribosomes^8^,^12^,^13^,^14^ suggest that aminoglycosides do so by binding the major groove of h44 to shift the position and dynamics of two universally conserved residues (A1492 and A1493) responsible for the recognition of the mRNA codon-aminoacyl–tRNA (aa–tRNA) complex^2^,^15^.

While these investigations have greatly advanced our understanding of the molecular determinants of ribosome binding, they fall short of elucidating how the aminoglycosides affect each stage of the translation mechanism—initiation, elongation, termination and recycling^3^,^5^,^16^,^17^,^18^,^19^. They are also unable to fully explain how subtle chemical distinctions between structurally related aminoglycosides give rise to unique inhibition and resistance profiles^20^,^21^,^22^. Furthermore, a miscoding mechanism of action is at odds with observations that bacterial strains harbouring error-prone ribosomes are viable^23^,^24^ and evidence that specific aminoglycosides inhibit protein synthesis while exhibiting little, to no, miscoding^25^. A comprehensive understanding of aminoglycoside action on the ribosome must provide a rationale for these observations, as well as data suggesting that 2-deoxystreptamine aminoglycosides may operate through more than one functional site on the bacterial ribosome^18^,^21^,^26^.

Crystal structures of intact 70S *Escherichia coli* (*E. coli*) ribosomes have recently revealed that aminoglycosides bind to sites in both the small and large (50S) subunit of the bacterial ribosome^1^,^19^,^27^. We have shown that neomycin, the most potent and toxic member of the 4,5-linked aminoglycoside class^21^ (Fig. 1a), promotes translational miscoding and shutdown by simultaneously binding to the h44-decoding site and the major groove of H69 within the 30S and 50S subunits, respectively^1^ (Fig. 1b). These conserved rRNA elements, which comprise the central intersubunit bridge B2, undergo marked structural rearrangements during small-subunit rotation^1^, a process underpinning multiple aspects of the translation cycle^19^,^28^,^29^,^30^,^31^. It is not presently known whether, and to what extent, chemically related 4,5-linked aminoglycosides within the neomycin class impact the dynamics of subunit rotation.

Here, we present a detailed examination of structure–function relationships in chemically related 4,5-linked aminoglycosides to reveal that neomycin and paromomycin, which differ by only a single functional group, bind h44 and H69 to drive subunit rotation in opposite directions. These data, which include an analysis of both ribosome- and drug-resistance mutations, indicate that the distinct impacts of aminoglycosides on the collective motions of bridge B2 play a central role in their mechanisms of action. By solving the structure of the paromomycin–ribosome complex to 3.1 Å resolution, we show that such distinctions hinge on interactions of paromomycin with the apical tip of H69 via its 6′-hydroxyl moiety from within its canonical h44-binding site. These findings provide compelling evidence that mechanisms describing the potency and selectivity of this important class of anti-infective agents must include their capacity to alter the dynamic properties of bridge B2 via interactions with H69 of the large subunit.

## Results

**Figure 1:**
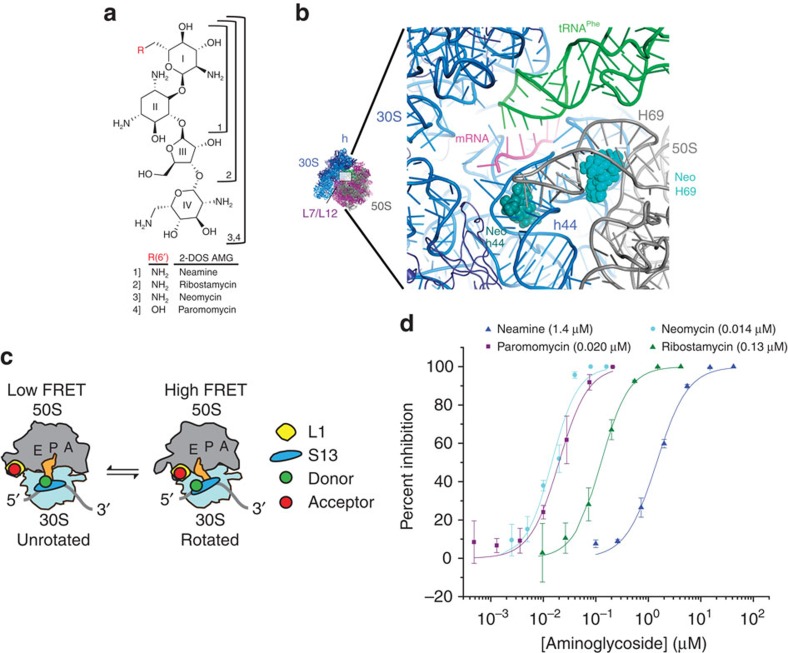
Investigation of the neomycin family of aminoglycosides. (**a**) 4,5-linked 2-deoxystreptamine (2-DOS) aminoglycosides in the neomycin family. (**b**) Overview of neomycin-binding sites in the small-subunit decoding site (h44, dark green) and in the large subunit H69 (light blue). 16S rRNA (30S) is shown in blue, 23S rRNA (50S) in grey, P-site tRNA in green and mRNA in magenta. (**c**) Cartoon illustrating the ribosome labelling strategy used for monitoring intersubunit rotation via single-molecule FRET, which has previously been shown to be affected by neomycin^1^. (**d**) Aminoglycoside-induced inhibition of *in vitro* translation. IC_50_ values are indicated. Experiments were performed in triplicate and the mean±s.d. is plotted.

We investigated the structural and dynamic impacts of 4,5-linked aminoglycoside antibiotic (Fig. 1a) binding to bridge B2 (Fig. 1b) within the intact *E. coli* 70S ribosome via single-molecule FRET (smFRET) by site-specifically labelling ribosomal proteins S13 and L1 within the small and large subunits with donor and acceptor fluorophores, respectively^1^ (Fig. 1c). To enable quantitative smFRET measurements of subunit rotation dynamics^32^,^33^,^34^,^35^, we employed intra-molecularly stabilized donor and acceptor fluorophores, which exhibit markedly enhanced brightness and photostability^36^ (see the Methods section). Using this system, smFRET data could be acquired at ∼2.6-fold faster time resolution than previously reported at an improved signal-to-noise ratio, where a total of ∼500 photons could be detected for each individual ribosome complex per imaging frame.

Coupled *in vitro* transcription–translation assays using purified translation components (see Methods) showed that ribosomes site-specifically labelled on proteins S13 and L1 were fully functional in processive translation reactions (Supplementary Fig. 1). This system was also employed to validate that neomycin and the chemically related 4,5-linked aminoglycosides paromomycin, ribostamycin and neamine (Fig. 1a) inhibit the function of our purified 70S *E. coli* ribosomes at concentrations consistent with those reported using S30 or S100 extracts^37^,^38^. Notably, neomycin and paromomycin exhibited roughly equivalent half-maximal inhibitory concentration (IC_50_) values (14–20 nM), whereas ribostamycin and neamine exhibited substantially reduced potencies (130 and 1,400 nM, respectively) (Fig. 1d). At face value, these data suggest that neomycin and paromomycin operate through similar mechanisms, where rings III and IV contribute as much as 100-fold to the overall affinity of the drug–ribosome interaction^7^,^39^.

### Native ribosomes exhibit spontaneous subunit rotation

**Figure 2:**
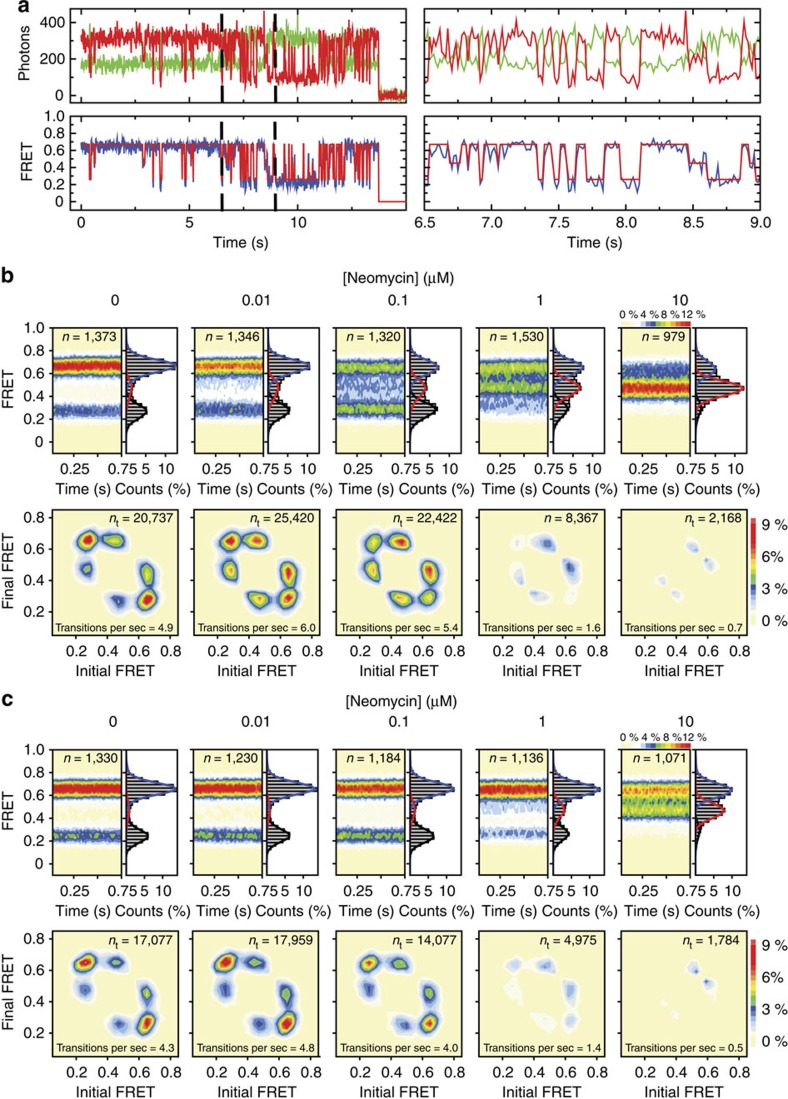
Neomycin-induced effects on intersubunit rotation in wild-type and A1408G aminoglycoside-resistant ribosomes. (**a**) (Left) Single-molecule fluorescence (donor—green; acceptor—red) and FRET (blue) trajectories illustrating typical conformational changes in ribosomes labelled as shown in Fig. 1c imaged in the absence of drug. FRET idealization is overlaid in red. (Right) A zoomed-in view highlights the transient nature of the intermediate-FRET state. (**b,c**) (top panels) smFRET trajectories summed into FRET histograms reveal the population behaviours across a range of neomycin concentrations in (**b**) wild-type and (**c**) A1408G ribosomes. (Bottom panels) Initial and final FRET values for each transition summed into two-dimensional histograms (transition density plots). Experiments were performed in triplicate on three separate days.

To explore the impact of distinct 4,5-linked aminoglycosides on the structure and dynamics of intact ribosomes, site-specifically labelled 70S complexes bearing deacylated tRNA^fMet^ in the P-site were imaged in the absence and presence of specific concentrations of 4,5-linked aminoglycosides. Such complexes spontaneously interconverted between high-FRET (0.67±0.01) and low-FRET (0.26±0.01) states on the sub-second timescale. Although these sites of labelling are in highly mobile elements of the ribosome—the L1 stalk^33^,^34^,^40^,^41^,^42^ and head domain^43^,^44^—dynamic FRET events principally reflect reversible transitions between ‘rotated’/’hybrid’ and ‘unrotated’/’classical’ ribosome configurations in which the S13 and L1 proteins are proximal and distal to each other, respectively^1^ (Figs 1c and 2a; Supplementary Fig. 2). This interpretation was further corroborated by imaging the process of subunit rotation from two distinct structural perspectives (Supplementary Figs 3 and 4).

Hidden Markov modelling-based idealization^45^ of the S13–L1 data obtained using the QuB software package (see the Methods section) also revealed evidence of a third, intermediate-FRET (0.45±0.01) state that appeared to be on path between low- and high-FRET configurations (Fig. 2a; Supplementary Fig. 5). The existence and kinetic linkage of these FRET states was corroborated by generating transition density plots from the idealized data^46^, which revealed six well-defined peaks symmetrically distributed with respect to the diagonal axis (Supplementary Fig. 5). Such findings suggest that at least one transient intermediate state of subunit rotation is transited on the path between unrotated and fully rotated ribosome configurations^1^.

### Steady-state actions of neomycin on intact 70S ribosomes

Ribosome complexes bearing deacylated tRNA^fMet^ in the P-site exhibited a pronounced bimodal response to the addition of neomycin^1^ (Fig. 2b; Supplementary Fig. 6a). Consistent with h44 binding^1^,^18^, low neomycin concentrations (up to 100 nM) increased the occupancy of the low-FRET, unrotated ribosome configuration by ∼1.4- to 1.6-fold (Fig. 2b; Supplementary Fig. 6a). The improved time resolution and signal-to-noise ratio afforded by the use of intra-molecularly stabilized fluorophores also revealed that neomycin concomitantly stabilized the intermediate-FRET state by ∼2.5-fold. At concentrations above 100 nM, the intermediate-FRET, partially rotated ribosome configuration was preferentially stabilized, accompanied by an approximately fivefold decrease in the global rate of intersubunit dynamics (0.7 versus 4.9 s^−1^ or 5.4 s^−1^) (Fig. 2b). These effects have been specifically attributed to neomycin’s interaction with the major groove of H69, which blocks the axial compression of H69 required for subunit rotation^1^. Hence, the predominant impact of neomycin binding to both h44 and H69 is to destabilize the unrotated ribosome configuration in favour of intermediate states of subunit rotation. Having established how ribosomes quantitatively respond to neomycin, we next set out to examine whether these changes in dynamics were altered by known drug-resistance modifications.

Consistent with previous investigations of resistance modifications to neomycin^21^,^47^, we observed increased IC_50_ values for both the ring-I-modified, 6′-*N*-acetyl-neomycin (0.75 μM) and the ring II-modified 3-*N*-acetyl-neomycin (34 μM) (Supplementary Fig. 7; Supplementary Table 1). In agreement with the mode of interaction between neomycin and the ribosome observed crystallographically^1^ in which rings I and II of the drug recognize chemical features within the major groove of both h44 and H69, subunit rotation dynamics were only weakly affected (1.4–2-fold) by 6′-*N*-acetyl-neomycin and largely unaffected by 3-*N*-acetyl-neomycin (see the Methods section; Supplementary Fig. 8). 6′-*N*-acetyl-neomycin modestly stabilized (twofold) both low- and intermediate-FRET states at concentrations above 1 μM (Supplementary Fig. 8). By contrast, 3-*N*-acetyl-neomycin exhibited little, to no, detectable impact on the FRET-state distribution even at the highest concentrations tested (10 μM; Supplementary Fig. 8).

The contributions of rings III and IV of neomycin to the observed impact on subunit rotation dynamics and processive translation were examined through analogous investigations of the closely related 4,5-linked aminoglycosides, ribostamycin (rings I–III) and neamine (rings I and II) (Fig. 1a). Consistent with previous literature^7^,^20^,^37^ and the crystallographically observed modes of interaction of neomycin with h44 and H69 (ref. 1), both ribostamycin and neamine exhibited substantially reduced impacts on subunit rotation dynamics and correspondingly higher IC_50_ values in coupled *in vitro* transcription–translation reactions (Supplementary Table 1). At the highest concentrations tested (10 μM), both drugs elicited low- and intermediate-FRET stabilization (Supplementary Fig. 6). Collectively, these findings reveal that rings I and II are necessary and sufficient to mediate drug binding to h44 and H69.

### The h44- and H69-binding sites are functionally linked

Clinical isolates resistant to the 4,5-linked aminoglycosides exhibit rRNA mutations that cluster in the h44-decoding site^2^. To investigate the impact of an h44 mutation on drug binding to the spatially separated h44- and H69-binding sites (Fig. 1b), identical experiments were performed on ribosome complexes bearing an A1408G point mutation that markedly reduces aminoglycoside binding to h44 (refs 7, 20) (Fig. 1b; see the Methods section) and the IC_50_ of *in vitro* transcription–translation (42 versus 0.014 μM) (Supplementary Fig. 7; Supplementary Table 1). Ribosomes bearing the A1408G mutation exhibited globally similar dynamic behaviours to wild-type ribosomes (Fig. 2b,c), and as anticipated from reduced binding at the h44-decoding site, the low-FRET, unrotated-state population did not increase in the presence of neomycin (Fig. 2c). A modest increase in intermediate-FRET state occupancy was evidenced only at elevated (>1 μM) neomycin concentrations that correlated with a global dampening of intersubunit rotation dynamics (Fig. 2c; Supplementary Fig. 9a). These distinctions are consistent with a functional linkage between the spatially separated neomycin-binding sites, where abrogation of drug binding at h44 concomitantly reduces the affinity and/or accessibility of the H69-binding site.

### Paromomycin stabilizes an unrotated ribosome configuration

To gain a deeper understanding of the contribution of ring I to 4,5-linked aminoglycoside interactions with the intact ribosome, we next set out to investigate the impact of paromomycin on wild-type and A1408G ribosomes. Paromomycin differs chemically from neomycin at a single position on ring 1: in neomycin, the 6′-position is a primary amine; in paromomycin, the 6′-position is a hydroxyl group (Fig. 1a). Despite their chemical similarities and their nearly indistinguishable IC_50_’s for the wild-type ribosome (Fig. 1d), neomycin and paromomycin exhibit unique sensitivities to resistance mutations in the decoding site^7^,^18^,^20^,^21^,^22^. Such distinctions have been attributed to subtle differences in their modes of interaction with specific bases within the h44 major groove^38^. Consistent with earlier studies, we observed that the A1408G mutation conferred substantially lower levels of resistance to paromomycin (IC_50_=1.6 μM) than for neomycin (IC_50_=42 μM) (Supplementary Fig. 7; Supplementary Table 1).

**Figure 3:**
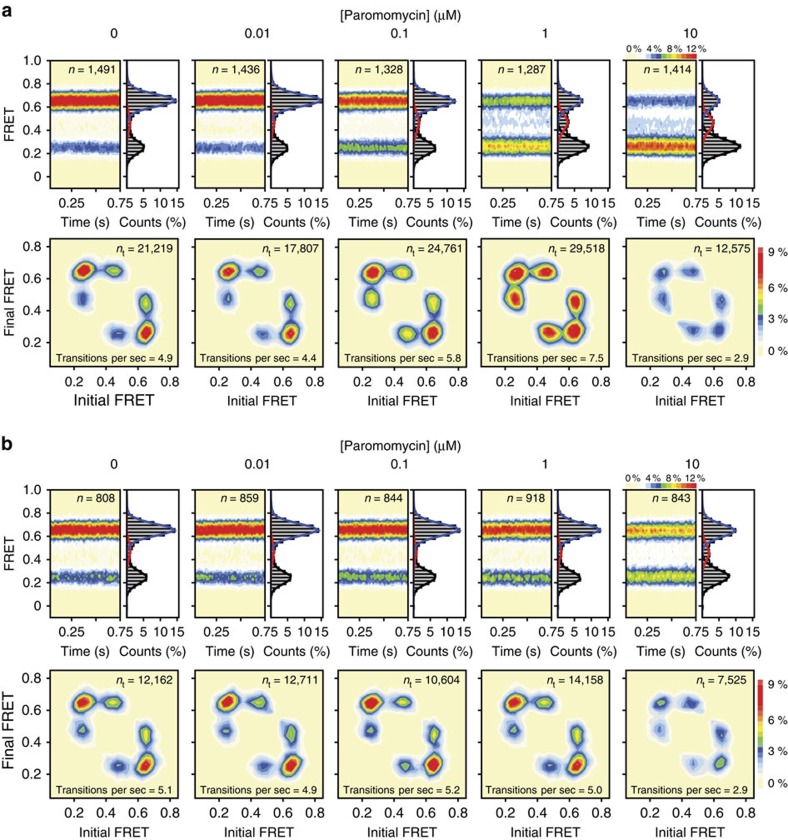
Paromomycin effects on intersubunit rotation in wild-type and A1408G aminoglycoside-resistant ribosomes. (**a,b**) (Top panels) smFRET trajectories summed into FRET histograms reveal the population behaviours across a range of paromomycin concentrations in (**a**) wild-type and (**b**) A1408G ribosomes. (Bottom panels) Initial and final FRET values for each transition summed into two-dimensional histograms (transition density plots). Experiments were performed in triplicate on three separate days.

Strikingly, and in line with its distinct impacts on the translation mechanism^21^, paromomycin was observed to preferentially stabilize the low-FRET, unrotated ribosome configuration at all concentrations tested (0.01–10 μM) (Fig. 3a; Supplementary Fig. 6). At concentrations up to 1 μM, such effects were notably correlated with a marked (∼100%) increase in subunit rotation dynamics (Fig. 3a, lower panels). Hence, despite paromomycin’s close chemical relation, its impacts are starkly opposite to those of neomycin. At concentrations above 1 μM, however, evidence of a modest stabilization of the intermediate-FRET state was observed; subunit rotation dynamics were also suppressed (Fig. 3a; Supplementary Fig. 6). Ribosomes bearing the A1408G mutation were largely unaffected by paromomycin up to a concentration of 1 μM, above which clear evidence of low- and intermediate-FRET state stabilization emerged, together with a concomitant dampening of intersubunit rotation dynamics (Fig. 3b; Supplementary Fig. 9b). Such findings, together with data obtained at lower pH (Supplementary Fig. 10), suggest that paromomycin’s distinct impacts on subunit rotation hinge on alterations in the drug’s interactions at both h44- and H69-binding sites and that these changes arise via the 6′-hydroxyl group and the drug’s net positive charge.

### Paromomycin contacts H69 from within the h44-binding site

**Figure 4:**
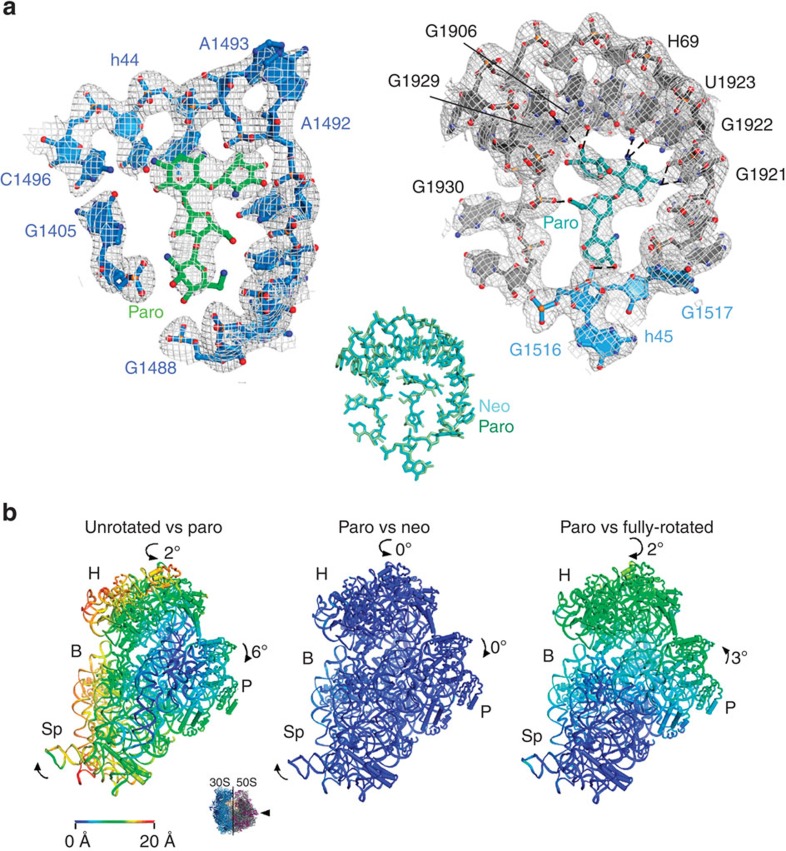
Crystal structure of the paromomycin-bound partially rotated ribosome. (**a**) Paromomycin binding within the (left) h44-decoding site and (right) within H69. 16S rRNA (blue), 23S rRNA H69 (grey) and paromomycin (h44—green; H69—teal) are shown, along with a (2*F*_obs_*−F*_calc_) electron density map, calculated in PHENIX and contoured at 1.4 s.d. from the mean. Paromomycin and H69 rRNA contacts <3.5 Å are shown as dashed lines. (Inset) Paromomycin contacts within H69 are indistinguishable from those formed by neomycin (r.m.s.d.=0.412 Å). (**b**) Paromomycin induces global rearrangements of the 70S ribosome that are indistinguishable from those stabilized by neomycin. (Inset) View of the 30S subunit from the perspective of the 50S subunit. (Left) Difference in the vector shifts between equivalent RNA phosphorus atoms and protein Cα atoms in the unrotated compared with the partially rotated paromomycin-bound ribosome; (middle) superposition of the partially rotated neomycin-bound and partially rotated paromomycin-bound ribosomes. (Right) The fully rotated compared with the partially rotated paromomycin-bound ribosome. The vectors are colour coded as indicated in the scale. Ribosomes were superimposed using the 50S subunit as the frame of reference. 30S head domain: H; 30S body: B; 30S platform: P; 30S spur: Sp.

To ascertain whether paromomycin’s distinct impacts on subunit rotation dynamics stem from unique modes of interaction with h44 and/or H69, we solved the crystal structure of the paromomycin–70S ribosome complex at a resolution of 3.1 Å (Table 1) under conditions enforcing drug protonation (below neutral pH) and intermediate-FRET state stabilization^1^ (see the Methods section; Supplementary Fig. 10). As observed for neomycin^1^, unbiased *F*_obs_*−F*_calc_ difference electron density maps revealed clear, positive electron density for paromomycin in the major grooves of both h44 and H69. To a first approximation, paromomycin’s modes of interaction with both sites appeared indistinguishable from those of neomycin (Fig. 4a), corroborating the placement and orientation of both 4,5-linked aminoglycosides within H69. The global conformations of both the neomycin- and paromomycin-bound ribosome were also indistinguishable. As observed for neomycin, and as suggested by the intermediate-FRET state value observed in the presence of both neomycin and paromomycin, the large and small subunits adopt a partially rotated configuration in which deacylated P-site tRNA is bound in an intermediate hybrid state (P/pe)^1^ (Fig. 4b; Supplementary Fig. 11).

**Figure 5:**
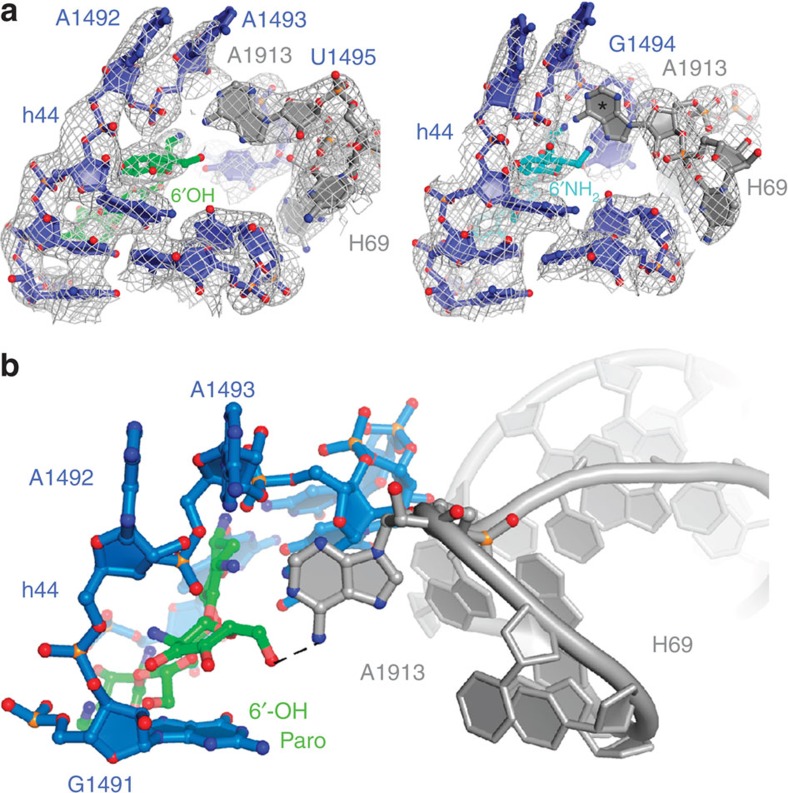
Paromomycin contacts the universally conserved A1913 residue of H69 from its canonical h44 site of binding. (**a**) The decoding site region of the paromomycin- (left) and neomycin- (right) bound ribosome (unrotated) exhibit strong and weak electron density for residue A1913 of H69, respectively. The 16S rRNA (blue), 23S rRNA (grey), h44-bound paromomycin (green) and h44-bound neomycin (light blue) are overlaid with feature-enhanced electron density maps^69^, calculated in PHENIX and contoured at 1.4 s.d. from the mean. The temperature factors for residue A1913 are as follows. Unrotated paromomycin-bound: ∼90 Å^2^; unrotated neomycin-bound: ∼430 Å^2^. The lack of density for A1913 in the neomycin-bound structure is highlighted with an asterisk. (**b**) Paromomycin (green) bound within the canonical small-subunit h44-decoding site (blue) contacts the apical tip of H69 via paromomycin ring I (6′-O) and the universally conserved base A1913 (N6) of H69 (grey).

However, one potentially critical distinction was observed between the neomycin- and paromomycin-bound ribosome crystal structures. In the presence of paromomycin, strong electron density was observed for the universally conserved A1913 residue located at the apical tip of the large subunit H69, whereas this was not observed in the neomycin-bound structure (Fig. 5a; Supplementary Fig. 12). These data suggest that the A1913 residue remains dynamic when neomycin is bound^1^, but is relatively static when paromomycin is bound. Inspection of the paromomycin data revealed the 6′-hydroxyl moiety of the h44-bound paromomycin molecule to be in hydrogen bonding distance (∼2.9 Å) of the A1913 Watson–Crick face (Fig. 5b). This finding, together with the observation that subunit rotation dynamics are largely unaffected by a 6′-deoxy-paromomycin analogue^48^ (Supplementary Fig. 13), indicates that the contact between the 6′-substituent in paromomycin and the apical tip of H69 is a critical determinant that drives the preferential stabilization of the unrotated ribosome configuration. Given that residue A1913 is observed to directly contact the anticodon loop region of tRNA bound within the A-site-decoding region^13^,^49^, the role of this contact on the aminoglycoside-induced miscoding mechanism warrants further exploration.

## Discussion

Functional investigations aimed at quantifying the impact of small-molecule inhibitors on the mechanism of protein synthesis face significant challenges when examined in the context of full-length protein production. This is due to the fact that mechanisms of action may be masked by additive or synergistic effects on multiple steps of protein synthesis. Inhibition of functional protein production may arise through impacts on any aspect of the system including translation, transcription, protein folding or by promoting protein inactivation or non-specific aggregation. Our examination of the 4,5-linked aminoglycosides in reconstituted protein translation reactions using purified components revealed direct evidence of hidden and complex modes of inhibition, as each of the 4,5-linked aminoglycosides examined, with the exception of neamine, exhibited IC_50_ values (14–130 nM) that were lower (2–18-fold) than the ribosome concentration in the reaction (250 nM). Moreover, both neomycin and paromomycin were observed to exhibit nearly indistinguishable IC_50_ values (Fig. 1d; Supplementary Table 1) despite their apparently distinct impacts on the dynamics of subunit rotation (Figs 2 and 3). By contrast, isolated structural investigations provide only snapshots of a drug’s impact on its target. Indeed, our comparison of neomycin- and paromomycin-bound ribosome structures revealed just a single obvious distinction: the position and dynamics of residue A1913 (Fig. 5; Supplementary Fig. 12). These findings provide a compelling argument that single-molecule imaging methods offer a means to bridge these distinct approaches to generate insights into dynamic structural processes directly related to function.

The process of subunit rotation underpins essential aspects of the translation mechanism and entails large-scale, functional remodelling events within intersubunit bridge B2 (refs 1, 31). In line with cryo-electron microscopy investigations of the bacterial ribosome^50^, we find that the subunit rotation process is rapid and reversible under the present experimental conditions (ca. 5 s^−1^; see the Methods section). We have leveraged this inherent metastability and the synergies of smFRET and X-ray crystallography to gain a deeper understanding of the actions of chemically related aminoglycosides on the intact 70S ribosome. These data reveal that both neomycin and paromomycin bind the major grooves of both h44 and H69, central features of the bridge B2 element. Drug binding at these structurally distinct, functionally linked, sites impacts the nature and rate of intersubunit rotation dynamics in opposing ways. Binding to h44 stabilizes an unrotated ribosome configuration, whereas drug binding to H69 stabilizes an intermediate state of subunit rotation. Simultaneous binding at both sites secures the ribosome in an intermediate state of rotation that is incompetent for basal translation elongation reactions^1^. Together with the present investigations of chemically related 4,5-linked aminoglycosides and resistance modifications to both neomycin and the ribosome, we conclude that binding at both the h44 and H69 sites within bridge B2 contributes to the impact of 4,5-linked aminoglycoside antibiotics on protein synthesis.

The present observation of functional linkages between the h44- and H69-binding sites is likely a natural consequence of the collective conformational changes that occur within bridge B2 during subunit rotation. This conclusion warrants further examination, particularly as it relates to aminoglycoside mechanisms of action and toxicity^6^,^16^. While h44 sequences vary substantially between bacterial and human cells, the corresponding sequences of H69 are more closely related^51^. The extent of H69 sequence conservation and its functional role in the process of subunit rotation may explain why some aminoglycosides impinge upon the translation machinery of the host cells and why aminoglycoside-resistance mutations have yet to be reported within H69. Our own efforts to site-specifically alter the H69 sequence to confer aminoglycoside resistance have only led to modest reductions in neomycin binding when four nucleotides in the helical region of H69 were swapped to the corresponding human sequence. Notably, these mutations imparted a marked increase in subunit rotation dynamics and modestly stabilized the high-FRET, rotated ribosome configuration (Supplementary Fig. 14; see the Methods section). While more significant H69 mutations may be expected to confer greater aminoglycoside resistance, they are likely to be lethal when made in isolation. To best address this issue, a comprehensive, targeted screen for resistance mutations will need to be performed taking into account the consideration that bridge B2 exhibits collective conformational transitions that regulate the nature and rate of intersubunit rotation.

A molecular rationale that explains how the A1408G-resistance mutation in the h44-decoding site reduces the apparent impact of aminoglycoside binding to H69 (Figs 2c and 3b; Supplementary Fig. 9) is not immediately obvious. The insight provided by the paromomycin-bound ribosome crystal structure, that aminoglycosides located within the h44 major groove can directly contact the apical tip of H69, suggests that mutations that reduce h44 affinity may simultaneously alter the local geometry of bridge B2 in a manner that indirectly affects drug interactions with the H69 major groove. A reduced affinity for H69 may also arise from global changes in the accessibility of the H69-binding site related to compression of its major groove during subunit rotation^1^,^31^. For instance, the H69-binding site is sterically occluded when the ribosome adopts a fully rotated configuration and may only be optimally formed upon partial subunit rotation. In this view, small changes in the underlying energy landscape of subunit rotation may conformationally mask the H69 major groove by altering the effective concentration of the transient H69-binding site. Considerations of this kind may indeed contribute to aminoglycoside resistance in eukaryotes, as the 80S ribosome preferentially adopts rotated states^52^,^53^.

**Figure 6:**
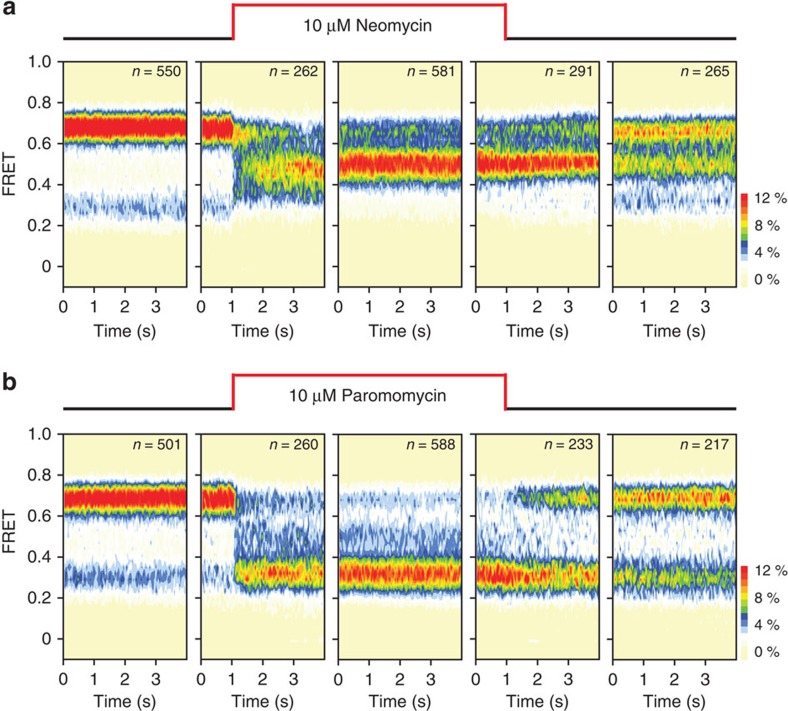
Neomycin and paromomycin rinse off of h44 but not H69. Aminoglycoside real-time deliveries and rinse-outs were performed on wild-type ribosomes. For both (**a**) neomycin and (**b**) paromomycin, five population FRET histograms are shown representing the chronological order of the experiment. Left to right: (1) no drug; (2) real-time delivery of 10 μM aminoglycoside; (3) aminoglycoside equilibrium; (4) real-time rinse-out; (5) 90 s following rinse-out.

Preliminary efforts to examine kinetic features of neomycin and paromomycin binding to the ribosome reveal that their on- and off-rates to both h44 and H69 must also be taken into consideration (Fig. 6). Neomycin rapidly and simultaneously binds the major grooves of both h44 and H69 to stabilize the partially rotated ribosome, whereas following buffer exchange, the distribution of states exhibited resemble those observed in the context of the A1408G mutation, in which residual binding at H69 persists (Figs 2c and 6a). By contrast, paromomycin appears to quickly bind (ca.<1 s) and preferentially stabilize the unrotated ribosome configuration while dissociating relatively slowly from its h44-binding site upon buffer exchange (Fig. 6b). These observations suggest that aminoglycosides reversibly bind the h44-decoding site^18^, whereas binding may be more avid to H69. Thus, distinct aminoglycoside actions in translation must include considerations about the disparities in the kinetic parameters of h44 and H69 binding. A quantitative understanding of aminoglycoside actions on the ribosome should therefore include considerations of the ribosome and drug concentrations, the dynamics of subunit rotation and the drug’s net charge. This latter point is particularly crucial, given that the cell’s proton gradient has been reported to dissipate upon aminoglycoside treatment^16^, an outcome that is expected to increase the avidity of drug binding to bridge B2.

The capacity to image aminoglycoside activities on intact ribosomes greatly extends our knowledge of the mechanisms of action of specific aminoglycosides beyond that which could be achieved using model oligonucleotide systems and offers a powerful approach for exploring drug actions on defined ribosome complexes. However, the present investigations, together with observations that conformational processes in the ribosome are context dependent^18^, suggest that the impact of aminoglycosides may be non-uniform in translation. Thus, targeted investigations probing the many distinct ribosome complexes transited during processive translation reactions must be further explored. A complete understanding of aminoglycoside actions in the cell may ultimately depend on the marriage of technological developments affording increased imaging throughput with those providing global snapshots of ribosome positions on specific mRNA species during active translation^54^.

## Methods

### Reagents

Neomycin trisulfate salt hydrate (Sigma-Aldrich), paromomycin sulfate salt (Sigma-Aldrich), ribostamycin sulfate salt (Sigma-Aldrich), neamine hydrochloride (Toronto Research Chemicals) and puromycin dihydrochloride (Sigma-Aldrich) were used at the purity stated by the commercial suppliers (≥97%). Chemoenzymatic preparation of acetylated forms of neomycin is described below. 6′-deoxy-paromomycin pentaacetate salt was synthesized by the Vasella group (ETH Zurich) as described^48^ and was a kind gift from David Crich (Wayne State University). All smFRET experiments were performed in Tris-polymix buffer (pH 7.5) containing 50 mM Tris acetate, pH 7.5, 5 mM Mg(OAc)_2_, 100 mM KCl, 5 mM NH_4_OAc, 0.5 mM CaCl_2_, 0.1 mM EDTA, 5 mM putrescine and 1 mM spermidine.

### Generation of site-specifically labelled ribosome complexes

30S subunits enzymatically labelled on S13 (N-Sfp) with LD550 (Lumidyne Technologies) and 50S subunits labelled with LD650 on L1 (T202C) were prepared and purified as previously described^1^,^40^. To label ribosomal protein L5 on native ribosomes, L5 was PCR cloned from *E. coli* strain K12 genomic DNA into the pPROEX HTb vector with a TEV-protease-cleavable histidine (His)_6_ tag and a 12-residue peptide encoding the A1 epitope for the AcpS phosphopantetheinyl transferase reaction^55^ (amino-acid sequence, GDSLDMLEWSLM) fused at the N terminus (N-AcpS). After plasmid shuffling into an *E. coli* ΔL5 knockout strain^56^, cells were cultured and ribosomes were harvested, cleaved and labelled *in situ* as performed for S13-labelled subunits^1^. Cy5-fMet-tRNA^fMet^ (Cy5-s^4^U8) was purified^57^ and labelled^58^ as previously described.

The A1408G point mutation in h44 of 16S rRNA was introduced into *in vivo* assembled ribosomes containing N-Sfp S13 using the previously described MS2 tag protocol^59^ (pSpurMS2). Similarly, the H69 mutations (C1908G, C1909A, G1921U and G1922C) in 23S rRNA were introduced into *in vivo* assembled ΔL1 ribosomes^40^ using the MS2 tag protocol^59^ (p278MS2). To form intact 70S ribosome complexes, LD550-S13 30S and LD650-L1 50S subunits were heat activated at 42° C for 10 min in Tris-polymix Mg^2+^ buffer. Ribosomes were then initiated with fMet-tRNA^fMet^ as previously described^60^.

### Acquisition and analysis of subunit rotation dynamics

smFRET experiments were performed using a prism-based total internal reflection microscope^45^,^60^ at 25 °C. Ribosome complexes programmed with biotinylated mRNA and bearing P-site fMet-tRNA^fMet^ were surface immobilized via a biotin–streptavidin bridge within polyethylene glycol (PEG)-passivated, streptavidin-coated quartz microfluidic chambers^45^,^60^ and imaged in Tris-polymix buffer with 5 mM Mg^2+^, supplemented with an oxygen scavenging system^61^ and triplet-state quenching compounds^62^ (1 mM cyclooctatetraene, 1 mM nitrobenzyl alcohol and 1 mM Trolox). Prior to experiments, P-site fMet-tRNA^fMet^ was deacylated by incubation with 2 mM puromycin, a mimetic substrate of the peptidyltransferase centre that liberates nascent peptide chains^63^, for 10 min at 25 °C in Tris-polymix buffer at pH 8.5. Aminoglycoside titrations were executed at pH 7.5, unless otherwise noted.

smFRET data were acquired by directly exciting the LD550 fluorophore linked to ribosomal protein S13 at 532 nm (LaserQuantum), while the LD550 and LD650 intensities were simultaneously recorded in Metamorph (Molecular Devices) at a 15-ms integration time. Using this approach, fluorescence and FRET trajectories could be obtained from hundreds of surface-immobilized, fluorescently labelled ribosome complexes simultaneously, where an average of 500 photons were collected for each ribosome complex per image frame. FRET traces were calculated as: FRET=*I*_LD650_/(*I*_LD550_+*I*_LD650_), where *I*_LD550_ and *I*_LD650_ are the instantaneous donor and acceptor fluorescence intensities, respectively. FRETing molecules were selected for analysis using custom-made analytical software implemented in MATLAB (MathWorks) using the following criteria: a single catastrophic photobleaching event, at least 8:1 signal-to-background-noise ratio and 6:1 signal-to-signal-noise ratio, less than four donor fluorophore blinking events, a correlation coefficient between donor/acceptor <0.5 and a lifetime of at least 50 frames (750 ms) in any FRET state ≥0.15. FRET trajectories were idealized to a four-state hidden Markov model (Supplementary Fig. 5) using the segmental k-means algorithm^45^. Occupancies in each FRET state were calculated from the idealized dwell times in each state divided by the total dwell time in all nonzero FRET states, and were plotted in Origin (OriginLab).

Prior to the puromycin reaction, ribosome complexes bearing fMet-tRNA^fMet^ in the P-site predominantly (∼80%) occupied a stable, low-FRET (0.26±0.01; mean-fitted FRET value±s.d. of the Gaussian centre over three biological replicates) state (Supplementary Fig. 2a). A battery of published biological controls^1^, together with high-resolution ribosome structures^1^,^31^, suggest that this FRET value corresponds to an ‘unrotated’/’classical’ ribosome configuration in which the S13 and L1 proteins are distal to each other (Fig. 1c). A small fraction (∼20%) of ribosomes exhibited high-FRET (0.67±0.01) (Supplementary Fig. 2a). This FRET value is consistent with a ‘rotated’/’hybrid’ configuration in which the S13–L1 inter-protein distance is substantially reduced (∼40 Å closer) (Fig. 1c). Consistent with this representing a subpopulation of ribosomes bearing deacylated P-site tRNA, all ribosomes were observed to exhibit a predominantly high-FRET state upon puromycin treatment (Supplementary Fig. 2b). Visual inspection of individual fluorescence and FRET trajectories obtained from puromycin-released complexes revealed time-dependent, anti-correlated fluctuations in donor and acceptor fluorescence intensities corresponding to low- to high-FRET state transitions (Fig. 2a). Consistent with the notion that P-site tRNA deacylation ‘unlocks’ the ribosome to enable the process of subunit rotation^42^, ribosome complexes bearing deacylated tRNA^fMet^ in the P-site spontaneously interconverted between unrotated and rotated configurations on the sub-second timescale. P-site tRNA deacylation increased the average transition rate between all three FRET states by ∼50-fold (∼4.9 versus ∼0.1 s^−1^) (Supplementary Fig. 5e). These data indicate that the observed motions predominantly report on transitions between unrotated, partially rotated and fully rotated configurations of the ribosome, rather than fast independent motions of the L1 stalk alone. Hence, we find that the process of subunit rotation in native ribosome complexes can occur at rates that are roughly an order of magnitude faster than EF-G-catalysed translocation^57^ and the rates previously reported through investigations of *in vitro* reconstituted ribosome particles^32^ or ribosomes hybridized to fluorescent oligonucleotides^35^. This metastability provided an ideal platform for exploring whether and to what extent chemically related aminoglycosides impact the ribosome’s energy landscape upon binding.

### Aminoglycoside association and dissociation experiments

The neomycin and paromomycin rinse-in/out experiments presented were performed using stopped-flow instrumentation on ribosomes labelled at L1–S13 and imaged as above, except the integration time was lowered to 40 ms to allow for a longer imaging window.

### Purification of 70S ribosomes for *in vitro* translation

Tight-coupled 70S ribosomes of a homogenous rRNA sequence were purified from strain SQ171 (Quan S., Skovgaard O. and Squires C.L., unpublished results) expressing the wild-type or A1408G rrnB operon using a previously described protocol^60^ with a slight modification. Prior to storage, ribosomes were buffer exchanged into 20 mM HEPES-KOH pH 7.8, 30 mM KCl, 10 mM Mg(OAc)_2_ and 1 mM TCEP-HCl using a 100-kDa MWCO Microcon (Millipore), and 1 molar equivalent of purified *E. coli* S1 protein was added. The ribosome/S1 mixture was then incubated at 37 °C for 10 min and cooled to 4 °C over ∼5 min. The solution was then exchanged into the same buffer and concentrated to [70S]>10 μM before flash freezing and storing in liquid nitrogen.

### Construction of the DNA template for *in vitro* translation

The DNA template used for *in vitro* protein synthesis reactions was a PCR product made by amplifying the *Photinus pyralis* luciferase gene. The primers were designed such that a T7 promoter followed by a bacterial Shine–Dalgarno sequence was added prior to the start codon and a T7 terminator sequence was added after the stop codon, as per the manufacturer’s recommendations (New England BioLabs).The primer sequences are listed below, where the firefly luciferase sequence (lower case letters), the T7 promoter (underlined), the Shine–Dalgarno sequence (bold) and the stop codon (italics) are indicated. Forward: 5′-GCGAATTAATACGACTCACTATAGGGCTTAAGTATA**AGGAGG**AAAAAATatggaagacgccaaaaacat-3′. Reverse: 5′-AAACCCCTCCGTTTAGAGAGGGGTTATGCTAG*TTA*cacggcgatctttccgccct-3′. Prior to use in coupled *in vitro* transcription–translation reactions, the PCR product was purified using the QIAquick PCR Purfication Kit (Qiagen) and resuspended in 0.5 × TE buffer to a final concentration of 50 ng μl^−1^.

### *In vitro* protein synthesis reactions

Solution A and Factor Mix were purchased from New England BioLabs as part of the PURExpress ΔRibosomes *In Vitro* Protein Synthesis Kit. Individual reactions contained 2 μl Solution A, 0.6 μl Factor Mix, 250 nM or 100 nM tight-coupled 70S ribosomes and 5 nM DNA template in a total volume of 5 μl. For the IC_50_ experiments, 4 μl of a reaction mix lacking aminoglycosides was added to 1 μl of aminoglycoside dilution in 0.2 ml thin-walled tubes on ice. For the ribosome activity assay, 4.1 μl of reaction mix was added to 0.9 μl of ribosomes or buffer on ice. Synthesis was initiated by incubation at 37 °C in a thermocycler (95 °C top) and terminated after 30 min by lowering the temperature to 4 °C for 10 min.

### Luciferase activity assay

Luciferase activity was quantified using the Luciferase Assay System (Promega). Fifty microlitre of luciferase assay reagent was added to the completed reactions, which were then transferred to white 96-well plates (Corning, 360 μl well volume) for luminescence quantification by an Infinite M1000 Pro microplate reader (Tecan). To determine IC_50_ of aminoglycosides, luciferase activity assays were performed in triplicate across a series of drug concentrations. A no-drug control (100% activity) was used to normalize luminescence measurements. Activity percentages were then converted to percent inhibition by subtraction from 100%. These values were then plotted on a semi-log scale versus aminoglycoside concentrations. To estimate IC_50_ values, plots were fit to the Hill equation: (100 × [aminoglycoside]^*n*^)/((IC_50_)^*n*^+[aminoglycoside]^*n*^).

### Chemoenzymatic preparation of 3- and 6′-*N*-acetyl-neomycin

The enzymes used to generate the *N*-acetyl-neomycin derivatives, AAC(3)-IV and AAC(6′)/APH(2″) were purified as previously reported^64^. Reaction mixtures (5 ml) containing trisulfate salt hydrate (5 mM, Sigma-Aldrich), acetyl coenzyme A lithium salt (7.5 mM, Sigma-Aldrich), ammonium bicarbonate (pH 7.0, 50 mM) and appropriate enzyme (0.1 mg ml^−1^ for AAC(6′)/APH(2″) and 0.03 mg ml^−1^ for AAC(3)-IV) were incubated with shaking at 37 °C (for AAC(6′)/APH(2″)) or 25 °C (for AAC(3)-IV). The reaction progress was monitored by thin-layer chromatography (TLC) (Merck, SiO_2_ gel 60 F_254_) using a 3:2/MeOH:NH_4_OH eluent system and staining with a cerium-molybdate stain ((NH_4_)_2_Ce(NO_3_)_6_ (5 g), (NH_4_)_6_Mo_7_O_24_·4H_2_O (120 g), H_2_SO_4_ (80 ml) and H_2_O (720 ml)). Reactions were generally complete after 24 h of incubation. Once the enzymatic conversion was completed, the enzyme was precipitated by addition of an equal volume (5 ml) of ice-cold MeOH. The mixture was cooled at −20 °C for at least 10 min and centrifuged (5,500 r.p.m., 10 min, 4 °C, Sorvall RC6 Plus centrifuge, rotor F21S-8x50) to pellet the protein. To extract any *N*-acetylated neomycin that could be associated with the protein, the protein pellet was resuspended in 1 ml of cold H_2_O, 1 ml of ice-cold MeOH was added and the precipitation process was repeated two more times. The 14 ml of combined solvent was removed *in vacuo* and the residue was resuspended in 1 ml of H_2_O. *N*-acetylated neomycin products were purified away from the CoA side-product on an Amberlite CG-50 (100–200 mesh: ammonium form, column packed=1 × 10 cm). The residual solution was loaded onto the ion-exchange column and washed with 15 ml H_2_O. The compounds were eluted stepwise (0.4% increments of 3 ml each for AAC(6′)/APH(2″) and 0.2% increments of 3 ml each for AAC(3)-IV) using a gradient of NH_4_OH (0–2%). The size of each fraction collected was 1.5 ml. To facilitate loading of the fractions onto the TLC plate for visualization, the fractions were diluted with 1.5 ml of MeOH. Fractions containing desired *N*-acetylated neomycin, as determined by TLC, were combined and the solvent was removed *in vacuo*. Both 6′-*N*-acetyl-neomycin and 3-*N*-acetyl-neomycin eluted between 0.4 and 0.8% NH_4_OH. Reactions yielded 13.1 mg of 3-*N*-acetyl-neomycin (80%) and 6.4 mg of 6′-*N*-acetyl-neomycin (39%). Note: the yield of the 6′-*N*-acetyl-neomycin is lower as it was utilized to optimize the purification protocol.

### Ribosome purification for crystallization

Ribosome purification from *E. coli* strain MRE600 was prepared as previously described^65^. Ribosome complexes with ribosome recycling factor (RRF) were made by incubating 2 μM ribosomes, 8 μM mRNA (5′-GGCAAGGAGGUAAAAUUCUACAAA-3′ Thermo Scientific) and 4 μM tRNA^Phe^ for 15 min at 37 °C. Next, 8 μM RRF was added and incubated for 15 min at 37 °C. Ribosomes were crystallized at 18 °C using microbatch 96-well plates and buffers containing 4.0–5.0% 2-methyl-2,4-pentanediol, 4.1–4.5% PEG 8000, 4.0 mM MgCl_2_, 380 mM NH_4_Cl, 5.7 mM putrescine, 5.0 mM spermidine, 10 mM Tris, 40 mM MES, pH 6.5–7.0 and 0.25 mM EDTA.

### Data collection and processing

Ribosome crystals were stepwise cryo-protected to the final conditions containing 7.0% 2-methyl-2,4-pentanediol, 7.0% PEG 8000 and 24% PEG 400, pH 4.8, to allow cryo-cooling of the crystals to liquid nitrogen temperatures. During the last cryo-protection step (PEG 400 24%), paromomycin sulfate salt (Sigma-Aldrich) was added at a 200-μM concentration to the cryo-protection buffer, and crystals were incubated at 4 °C with paromomycin. After 2 h of incubation, crystals were frozen with liquid nitrogen. Diffraction data were measured from crystals cooled to 100 K using 0.1–0.3° oscillations at the Advanced Light Source (beamlines 8.3.1 and 12.3.1), each of which is equipped with an ADSC Q315 area detector. Data were reduced using XDS^66^, yielding the statistics shown in Table 1.

### Molecular replacement and structure refinement

Molecular replacement and structure refinement was carried out in PHENIX^67^ platform, and previously published/deposited coordinates of neomycin-bound ribosomes (PDB ID: 4GAQ 4GAR 4GAS 4GAU) were used as a reference model^1^. Neomycin molecules were removed from the molecular replacement model to reduce possible model bias during refinement. Corrections to the resulting model were carried out in Coot^68^. The resulting structural models were then refined using rounds of manual rebuilding in Coot. Electron density maps were generated from the PHENIX output directly. RNA rebuilding concentrated on the tRNAs, H69 and h44 (neomycin-/paromomycin-binding sites) and paromomycin structures were inserted and refined using Coot and PHENIX. Paromomycin occupancies were determined by matching paromomycin atomic displacement parameters to the mean value of neighbouring rRNA, and then the group occupancy of each paromomycin was refined in PHENIX as previously described^1^. As with neomycin^1^, additional paromomycin-binding sites were found near A482 (H24), G551 (H25), G1157 (H41), U1240 (H46), A1858 (H68) and C2674 (H95) in 23S rRNA, and near C658 (h22), G902 (h27) and U1420 (h44) in 16S rRNA. Maps comparing bridge B2a in neomycin-bound (PDB ID 4V9C) and paromomycin-bound ribosome structures used feature-enhanced maps generated by phenix.fem in the PHENIX-1.9-1692 release, with default parameters plus the combined-omit map algorithm^69^.

### Superpositions

Superpositions were carried out in PyMOL using the 'pair_fit' command^70^. Disordered or flexible regions of 23S rRNA were not used (for example, the L1 stalk, the L7/L12 stalk, H38 and H69) in the superpositions. Superpositions were performed using ribose C1′ positions or phosphorus atoms in nucleotides. The angles of rotation of the 30S subunit domains were calculated essentially as described previously^1^,^31^. Angles given for the rotation of the head domain were calculated from 30S subunit structures superimposed by means of their platform domains. A rotation of 0° is defined as centering the head domain over the 30S P-site, as seen in the structure of the unrotated ribosome^31^. Superpositions of P/P, P/pe and P/E tRNAs used the C1′ atoms of nucleotides 31–39 in the anticodon stem loop^31^. The comparisons of tRNA bending angles used the glycosidic bond of position 31 near the end of the anticodon stem loop and the glycosidic bond of nucleotide 63 in superimposed tRNAs^31^. The calculated bending angles were 24° for P/P tRNA compared with P/pe tRNA, and 14° for P/pe tRNA compared with P/E tRNA. For the H69-bound neomycin and paromomycin rRNA comparison, 23S rRNA positions 1906–1909, 1919–1931 were used.

### Figure preparation

All structure figures were made using the program PyMOL^70^.

## Additional information

**Accession codes:** The structures reported here have been deposited in the Protein Data Bank with codes 4WOI, 4WON, 4WOM and 4WOO.

**How to cite this article:** Wasserman, M. R. *et al*. Chemically related 4,5-linked aminoglycoside antibiotics drive subunit rotation in opposite directions. *Nat. Commun.* 6:7896 doi: 10.1038/ncomms8896 (2015).

## Supplementary Material

Supplementary Information Supplementary Figures 1-14, Supplementary Table 1 and Supplementary References

## Figures and Tables

**Table 1:** Data collection and refinement statistics.

**Data collection**	
Space group	*P*2_1_2_1_2_1_
	
*Cell dimensions*	
* a*, *b*, *c* (Å)	212.10, 435.24, 614.44
Resolution (Å)*	70–3.1 (3.2–3.1)
CC1/2*	99.6 (42.5)
*I*/σI*	6.53 (0.68)
*R*_meas_*	17.8 (106.9)
Completeness (%)*	86.3 (54.7)
Redundancy*†	4.9 (1.7)
	
*Refinement*	
Resolution (Å)	70–3.0
Number of reflections	909,645
*R*_work_/*R*_free_	0.2141/0.2816
	
*Number of atoms*	
Protein/RNA	291,292
Ligand/ion	848
Water	1,729
	
*B*-factors	
Protein/RNA	64.3
Ligand/ion	61.6
Water	62.3
	
*R.m.s.d.*	
Bond lengths (Å)	0.009
Bond angles (°)	1.29

r.m.s.d., root mean squared deviation.

^*^Values in parentheses are for the highest-resolution shell.

^†^Data were measured from 16 crystals.
