# Supplementary material for: Chemically related 4,5-linked aminoglycoside antibiotics drive subunit rotation in opposite directions
Source: Nat Commun. 2015 Jul 30;6:7896. doi: 10.1038/ncomms8896 (PMC4522699; doi:10.1038/ncomms8896)
Supplement: Supplementary Information — Supplementary Figures 1-14, Supplementary Table 1 and Supplementary References [file ncomms8896-s1.pdf]

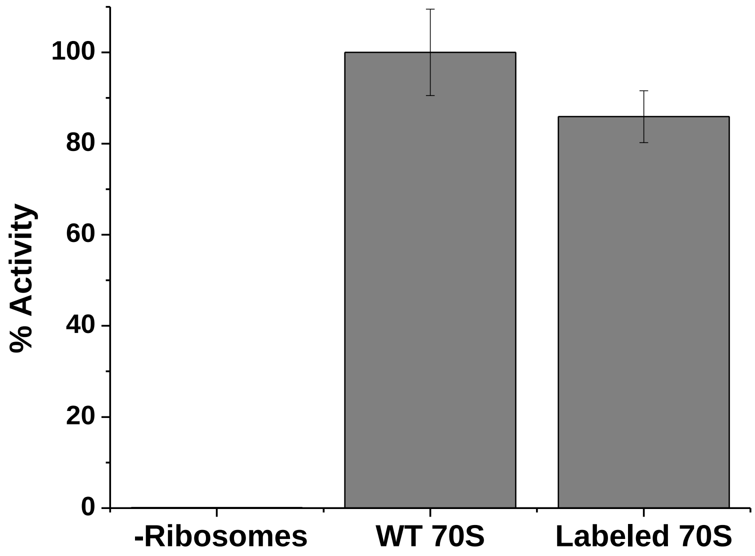

Supplementary Figure 1. Site-specifically labeled ribosomes are functional in processive translation reactions. Coupled *in vitro* transcription-translation assays performed with purified translation components (**Methods**) showed that site-specifically labeled ribosomes were fully functional in processive translation reactions. Wild-type 70S ribosomes were purified from *E. coli* strain SQ171 (**Methods**). Labeled ribosomes were prepared as indicated in the **Methods**. Error bars represent ± one standard deviation of a triplicate measurement.

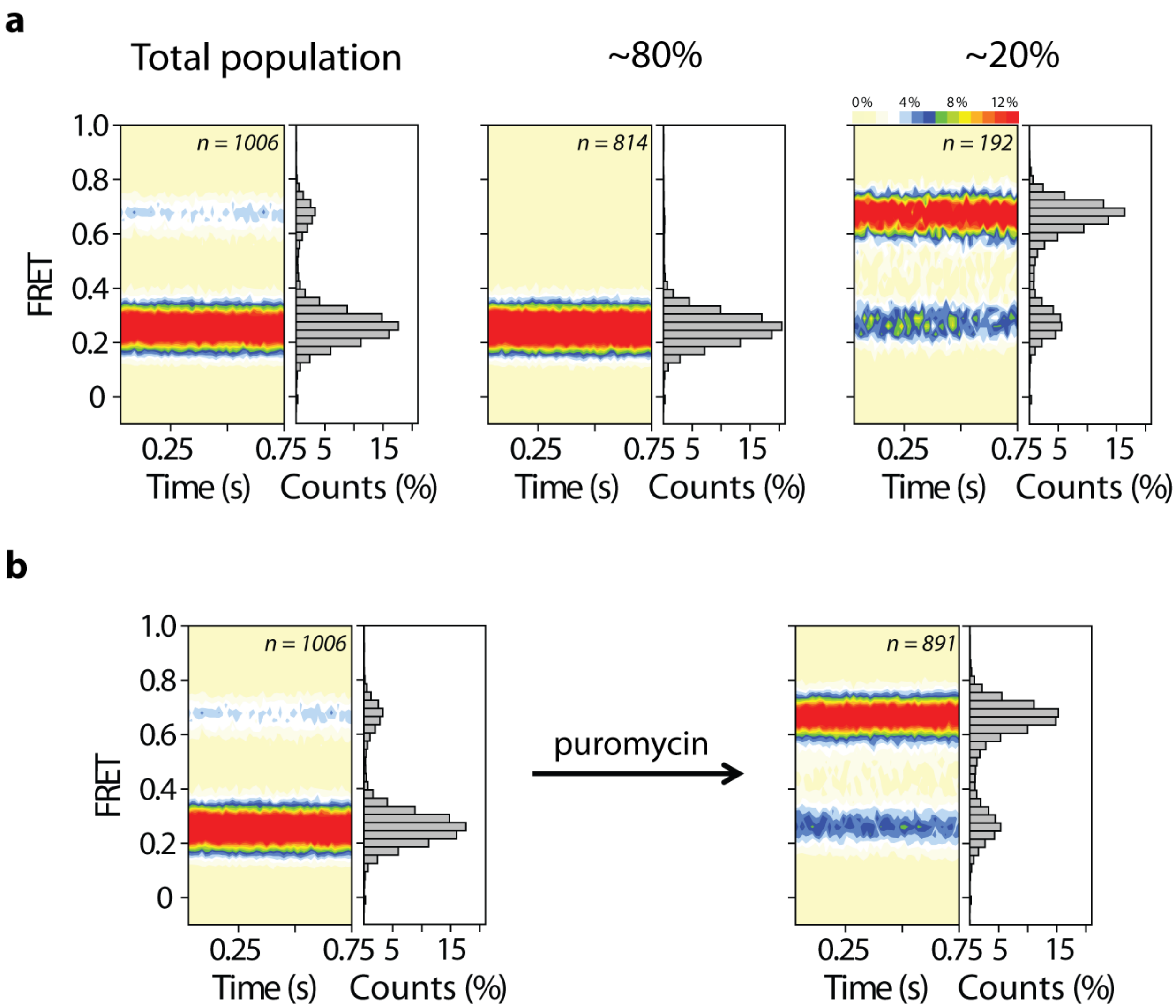

Supplementary Figure 2. Spontaneous intersubunit rotation is restricted by the presence of the formyl-methionine group on the P-site tRNA^{fMet}. (a) (left) As previously reported[1], S13-L1 labeled ribosome complexes bearing fMet-tRNA^{fMet} in the P site were observed to predominantly occupy a stable, low-FRET state consistent with an unrotated/"classical" ribosome configuration (**Figure 1c**) illustrated by summation of smFRET trajectories into FRET histograms. (center) The majority (~80%) of the population exhibited stable low-FRET; however, (right) approximately 20% of the population exhibited reversible fluctuations to a high-FRET state. (b) Puromycin treatment (**Methods**) efficiently converted the total population from (a) to a predominantly high-FRET state, suggesting that complexes exhibiting the dynamics shown in panel (a) (right) contained deacylated tRNA prior to puromycin treatment.

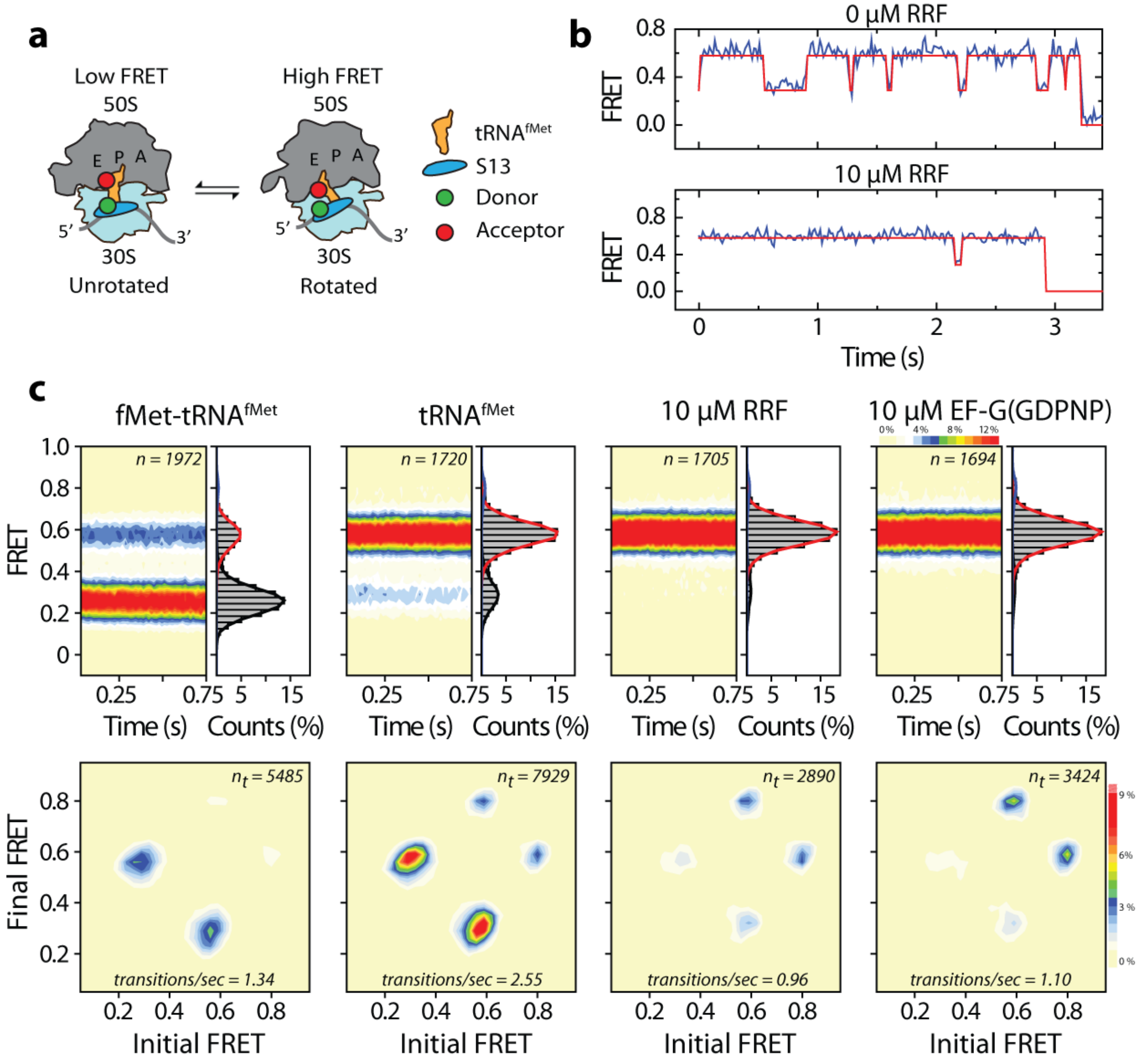

Supplementary Figure 3. FRET between ribosomal protein S13 and P-site tRNA^{fMet}. (a) Cartoon illustrating one of the ribosome labeling strategies used to provide an alternative structural perspective to S13-L1. (b) Representative single-molecule FRET trajectories illustrating typical conformational changes in ribosomes imaged in the (top) absence and (bottom) presence of RRF, which has been shown to stabilize the rotated configuration[1, 2]. FRET idealizations are overlaid in red (**Methods**). (c) As previously reported[1] and illustrated in **Supplementary Fig. 2**, ribosome complexes bearing fMet-tRNA^{fMet} in the P site were observed to predominantly occupy a stable, low-FRET state consistent with an unrotated/"classical" ribosome configuration illustrated by summation of smFRET trajectories into FRET histograms. Puromycin treatment (tRNA^{fMet} panel; **Methods**) efficiently converted the total population to a predominantly high-FRET state. Addition of saturating concentrations of RRF and EF-G(GDPNP) led to stabilization of the high-FRET, rotated state. Transition density plots (bottom panels), computed from hidden Markov modeling using a 3-state model (**Supplementary Fig. 5**), demonstrate that similar to the S13-L1 signal, dynamics increase upon treatment with puromycin and decrease upon the addition of RRF and EF-G.

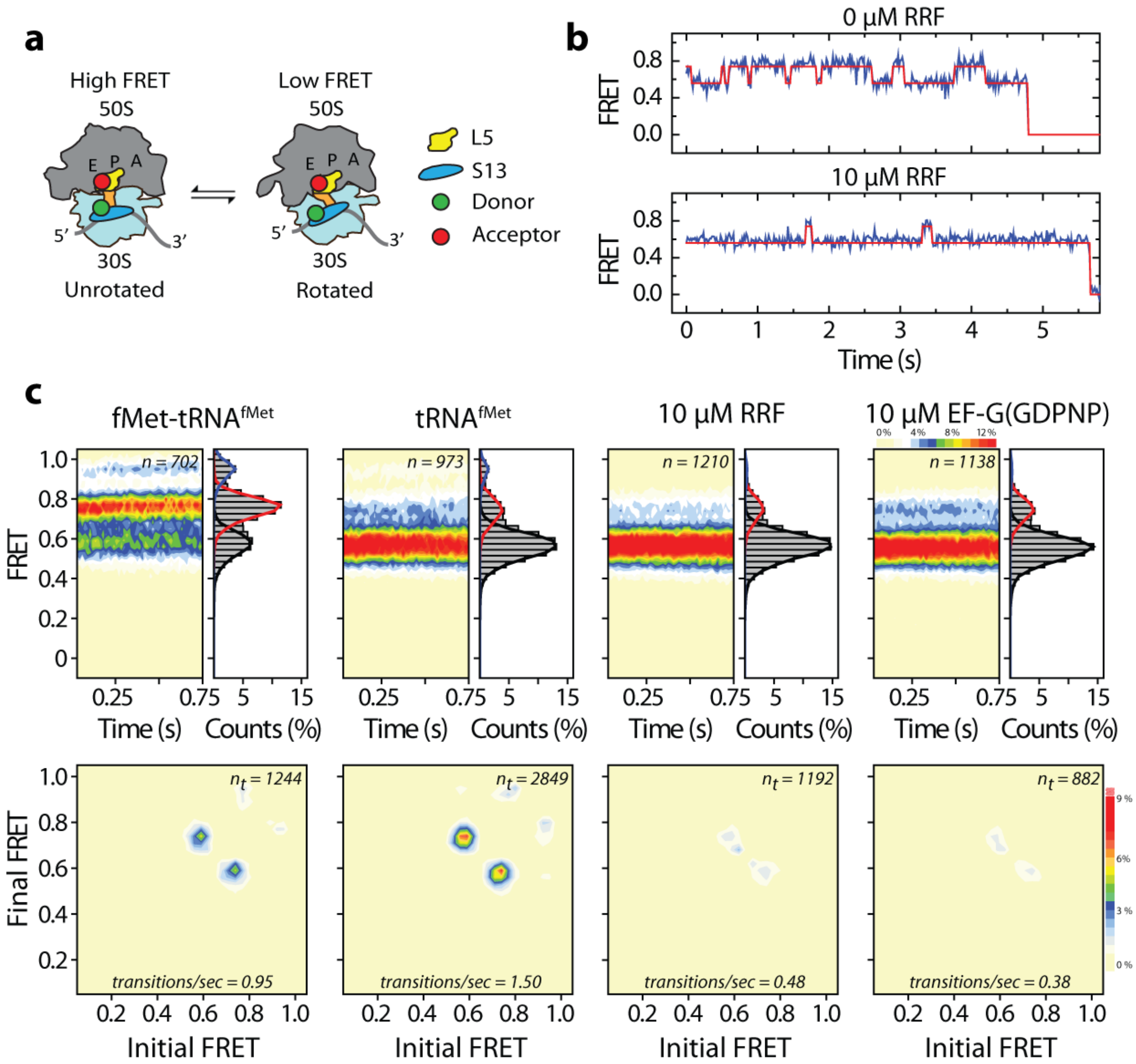

Supplementary Figure 4. FRET between ribosomal protein S13 and L5. (a) Cartoon illustrating one of the ribosome labeling strategies used to provide an alternative structural perspective to S13-L1. (b) Representative single-molecule FRET trajectories illustrating typical conformational changes in ribosomes imaged in the (top) absence and (bottom) presence of RRF, which has been shown to stabilize the rotated configuration[1, 2]. FRET idealizations are overlaid in red (**Methods**). (c) As previously reported[1] and illustrated in **Supplementary Fig. 2**, ribosome complexes bearing fMet-tRNA^{fMet} in the P site were observed to predominantly occupy a stable, high-FRET state consistent with an unrotated/"classical" ribosome configuration illustrated by summation of smFRET trajectories into FRET histograms. Puromycin treatment (tRNA^{fMet} panel; **Methods**) efficiently converted the total population to a predominantly low-FRET state. Addition of saturating concentrations of RRF and EF-G(GDPNP) led to stabilization of the low-FRET, rotated state. Transition density plots (bottom panels), computed from hidden Markov modeling using a 3-state model (**Supplementary Fig. 5**), demonstrate that similar to the S13-L1 signal, dynamics increase upon treatment with puromycin and decrease upon the addition of RRF and EF-G.

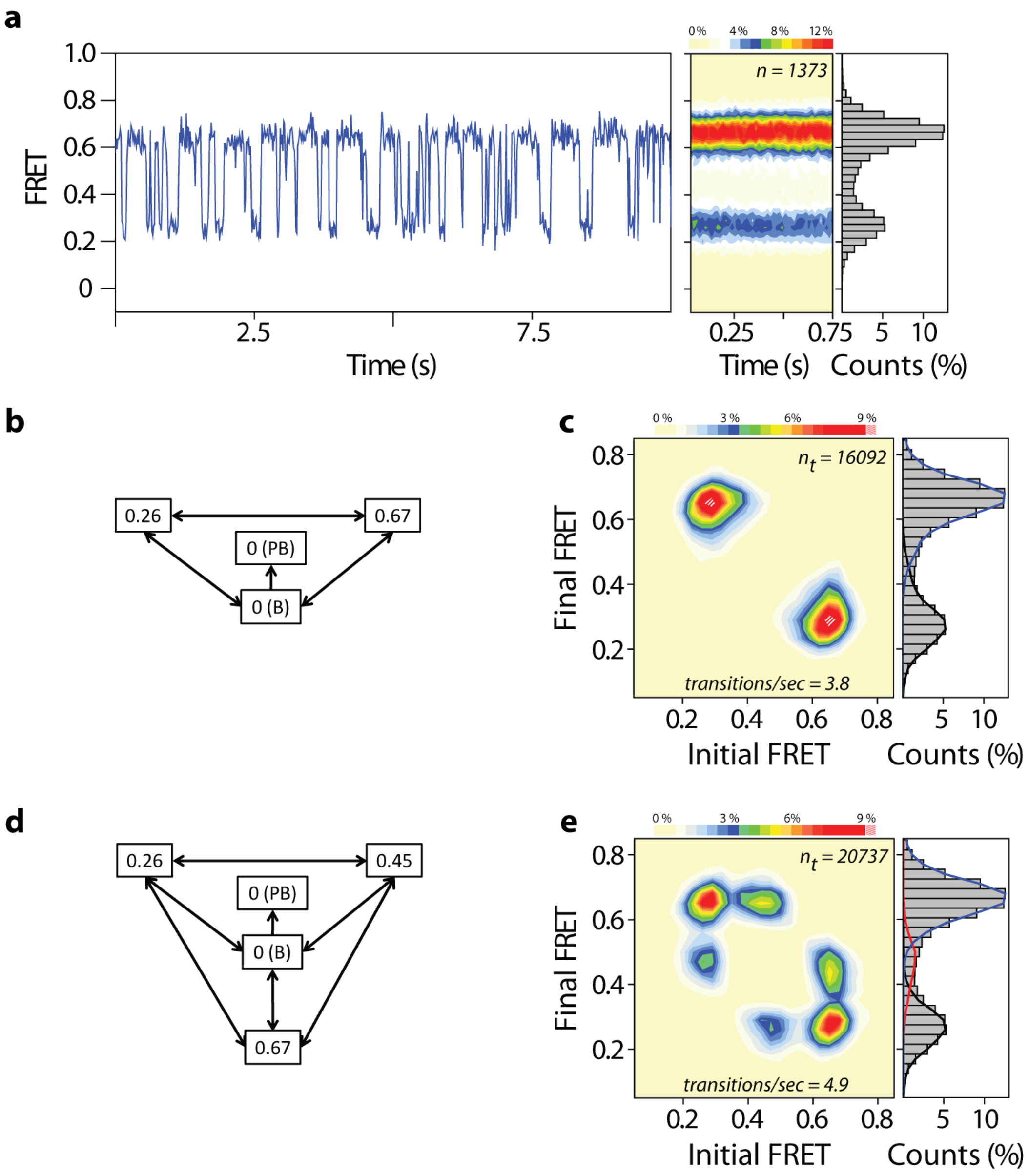

Supplementary Figure 5. Hidden Markov modeling of S13-L1 intersubunit rotation dynamics. (a) (left) A single-molecule FRET (blue) trajectory illustrating typical conformational changes in ribosomes labeled as shown in **Figure 1c** imaged in the absence of drug. (right) smFRET trajectories summed into a FRET histogram reveals the population behavior. (b) 2-state model used to idealize individual FRET trajectories *via* hidden Markov modeling (**Methods**) and the resulting (c) transition density plot. Here, initial and final FRET values for each transition summed into two-dimensional histograms show that transitions occur between at least two distinct FRET states. (d) 3-state model employed to idealize all S13-L1 traces in the current work (**Methods**) and the resulting (e) transition density plot, which suggests the existence of at least one short-lived intermediate FRET state. In both models, there are two zero-FRET state models; one for fluorophore blinking (B) and one for photobleaching (PB). The FRET idealization (black – 0.26; red – 0.45; blue – 0.67) is overlaid on the two-dimensional FRET histograms. Note that the same three-state model (with different FRET values) was used to idealize the S13-$tRNA^{fMet}$ and S13-L5 data.

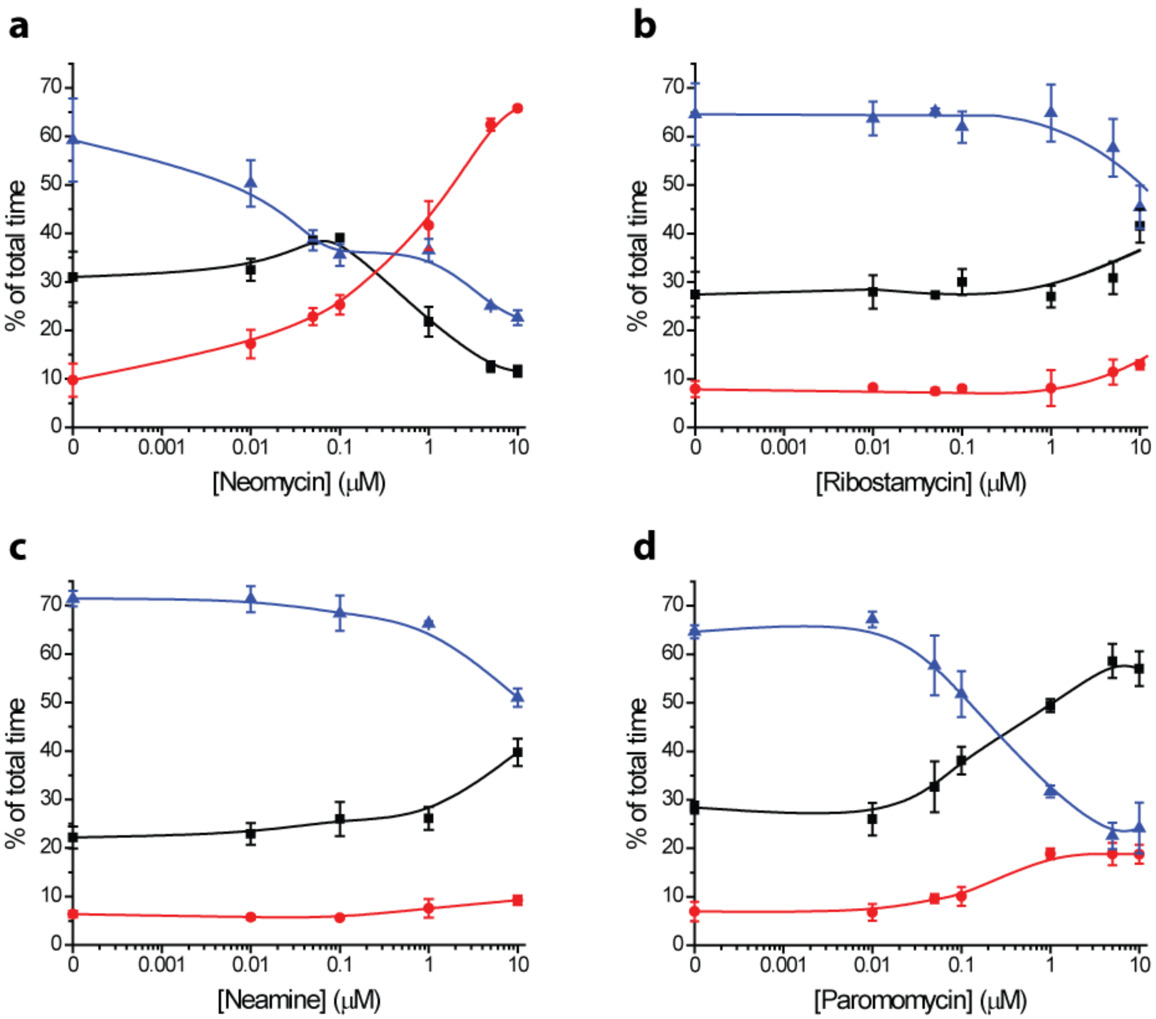

Supplementary Figure 6. 4,5-linked 2-deoxystreptamine aminoglycosides closely related to neomycin also affect intersubunit rotation. Titration summaries of (a) neomycin, (b) ribostamycin, (c) neamine and (d) paromomycin are plotted as percent time spent in each FRET state calculated from the idealized data (**Methods**). Low-FRET (black); intermediate-FRET (red); high-FRET (blue). B-spline functions are fit to the data to aid in visualization only. Experiments were repeated on either three (neomycin/paromomycin) or two (ribostamycin/neamine) separate days. The means ± standard deviations are plotted.

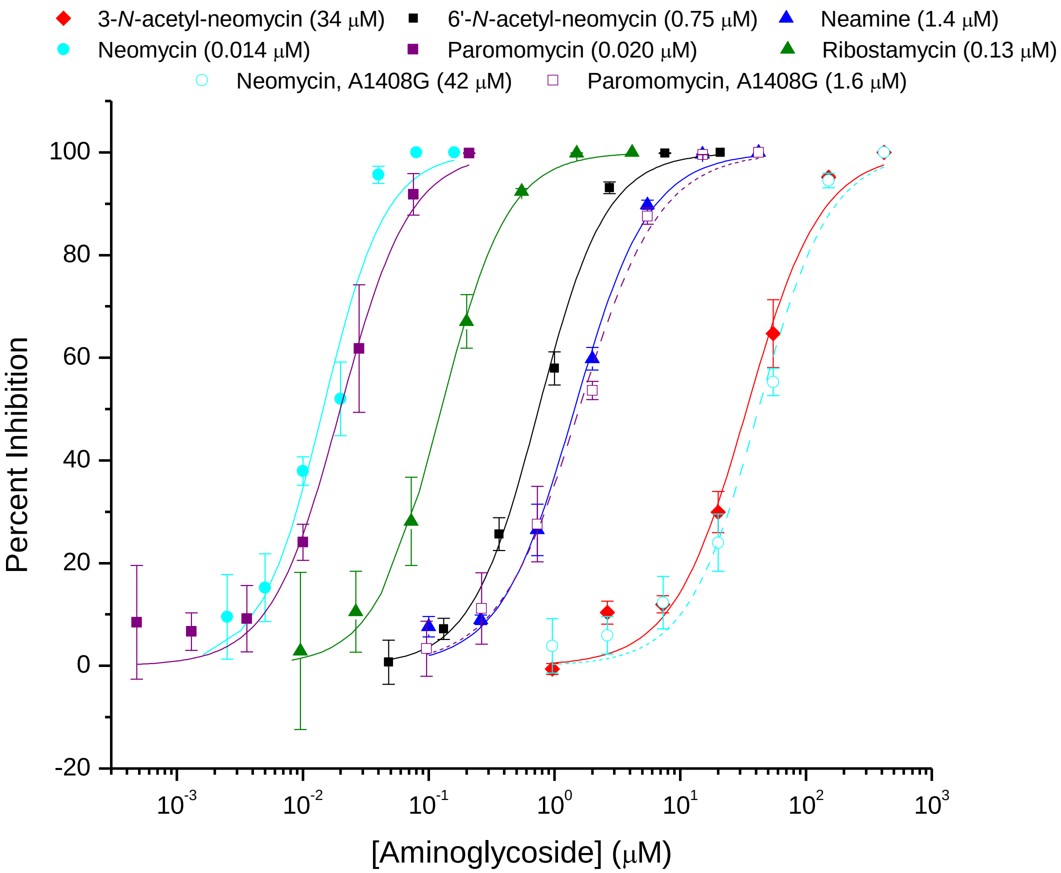

Supplementary Figure 7. Coupled *in vitro* transcription-translation assays were performed on wild-type and A1408G ribosomes in the presence of 4,5-linked 2-DOS aminoglycosides. The IC_{50} values are indicated. Assays were performed in triplicate and the means ± standard deviations are plotted. See **Methods** for a detailed description of the implementation and analysis of the assay.

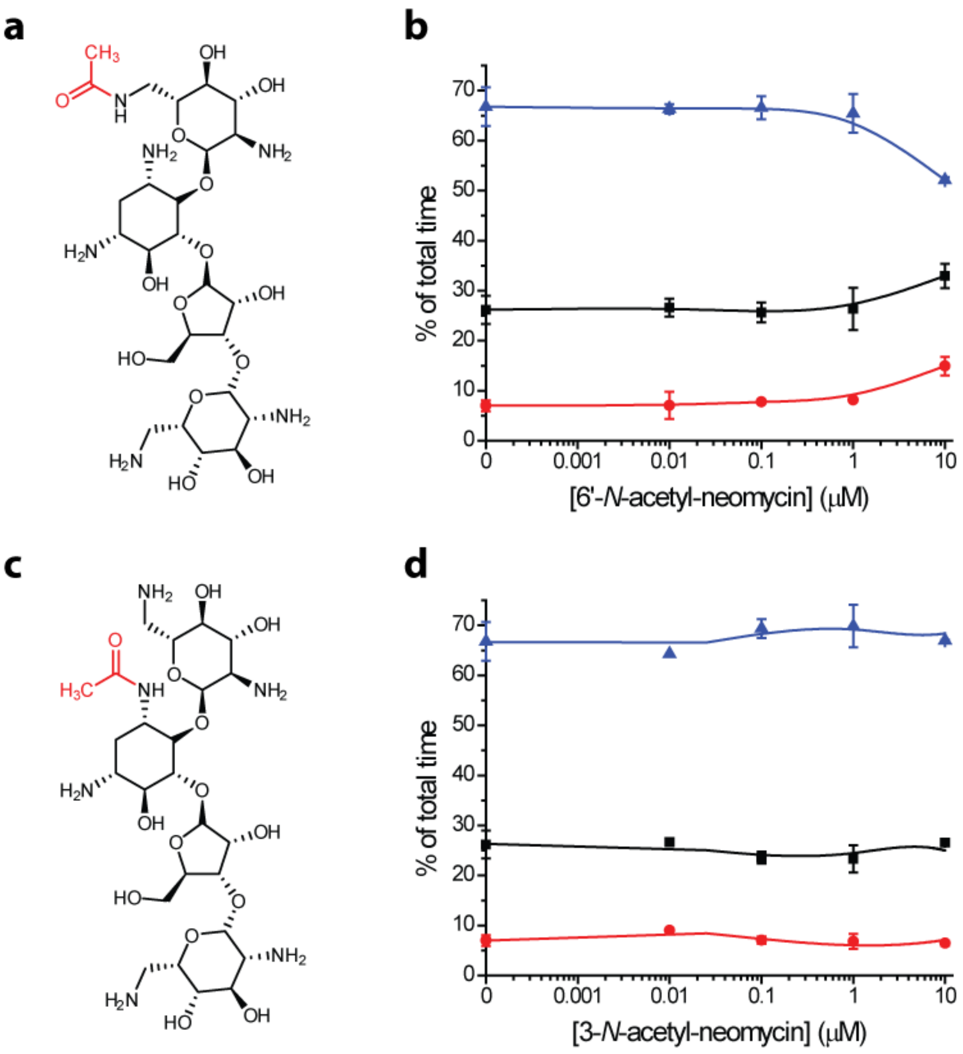

Supplementary Figure 8. Acetylated forms of neomycin known to confer resistance only weakly affect intersubunit rotation. (a) Chemical structure of 6'-*N*-acetyl-neomycin and (b) corresponding smFRET titration summary. (c) Chemical structure of 3-*N*-acetyl-neomcyin and (d) corresponding smFRET titration summary. Titrations are plotted as percent time spent in each FRET state calculated from the idealized data (**Methods**). Low-FRET (black); intermediate-FRET (red); high-FRET (blue). B-spline functions are fit to the data to aid in visualization only. Experiments were repeated on two separate days. The means ± standard deviations are plotted.

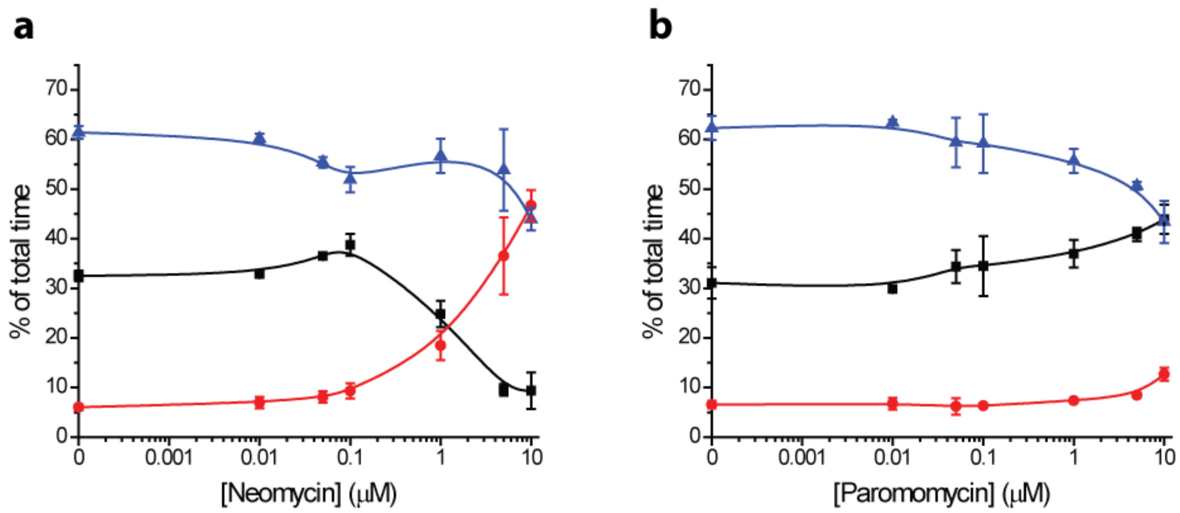

Supplementary Figure 9. Neomycin and paromomycin's effects on intersubunit rotation are attenuated in ribosomes containing an A1408G aminoglycoside resistance mutation. Titration summaries of (a) neomycin and (b) paromomycin on A1408G ribosomes are plotted as percent time spent in each FRET state calculated from the idealized data (**Methods**). Low-FRET (black); intermediate-FRET (red); high-FRET (blue). B-spline functions are fit to the data to aid in visualization only. Experiments were repeated on two separate days. The means ± standard deviations are plotted.

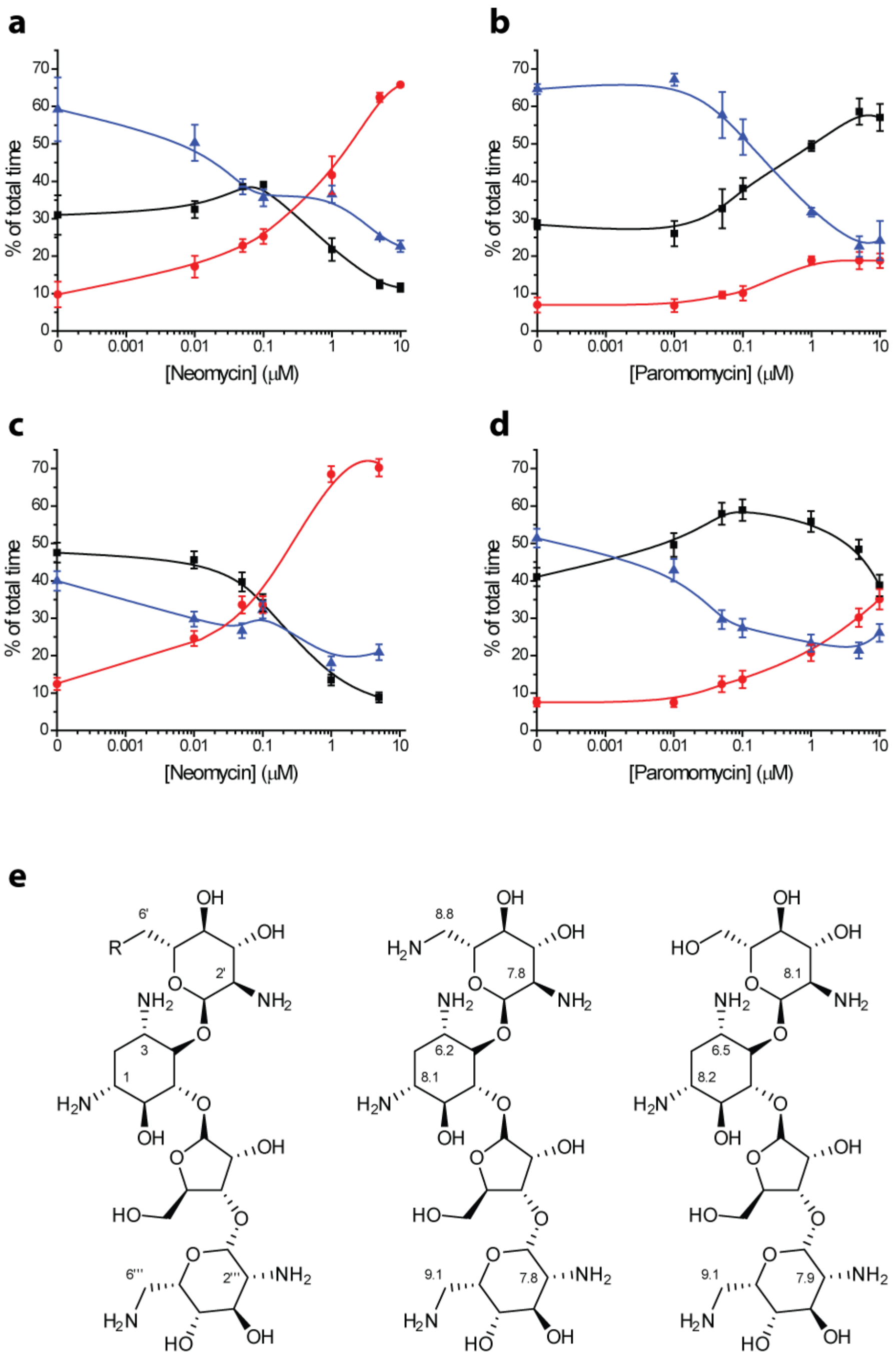

Supplementary Figure 10. Neomycin- and paromomycin-induced intermediate-FRET stabilization is enhanced at lower pH. Titration summaries are plotted as percent time spent in each FRET state calculated from the idealized data (**Methods**). (a) Neomycin and (b) paromomycin titration summaries at pH 7.5. (c) Neomycin and (d) paromomycin titration summaries at pH 6.5 (**Methods**). Low-FRET (black); intermediate-FRET (red); high-FRET (blue). B-spline functions are fit to the data to aid in visualization only. The means ± standard errors calculated from 10,000 bootstrap samples are plotted[3]. (e) (left) Chemical structure of neomycin (R=NH_2) and paromomycin (R=OH) with amine group position numbers indicated. Previously published pK_a values determined at 25 °C of (center) neomycin and (right) paromomycin are indicated[4, 5, 6].

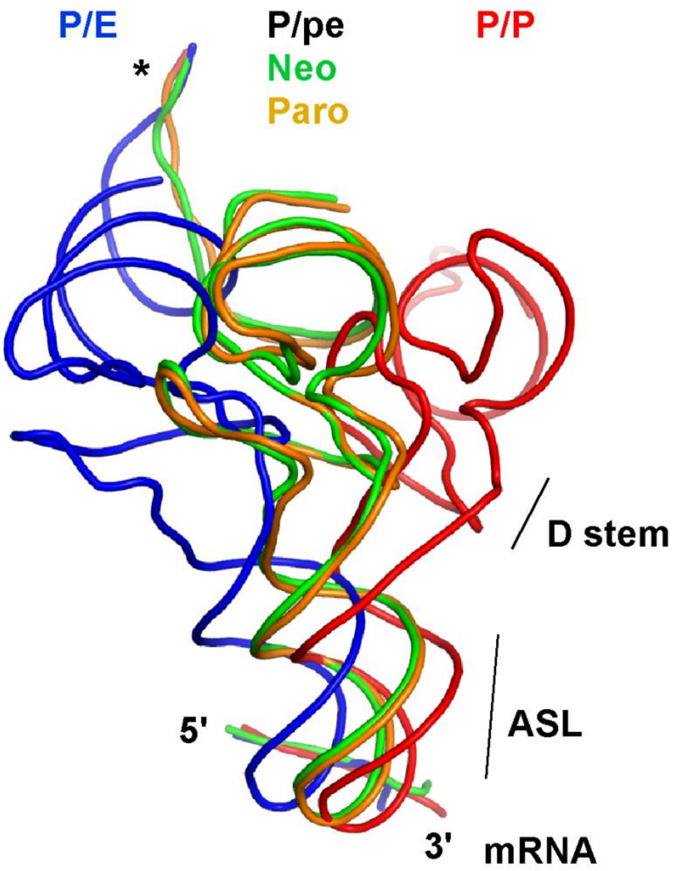

Supplementary Figure 11. Position of $tRNA^{Phe}$ in the intermediate-rotated paromomycin-bound ribosome. The intermediate position of the P-site tRNA (P/pe, gold) is very similar to that observed in the intermediate-rotated neomycin-bound ribosome[1] (green). In both cases, the P-site tRNA is positioned between the classical (P/P, red) and hybrid (P/E, blue) configurations in both the extent of T arm and anticodon displacement toward the E site. The 3' CCA end of P/pe tRNA (asterisk) occupies the large-subunit E site. The dihydrouridine (D) stem and anticodon stem loop (ASL) are indicated.

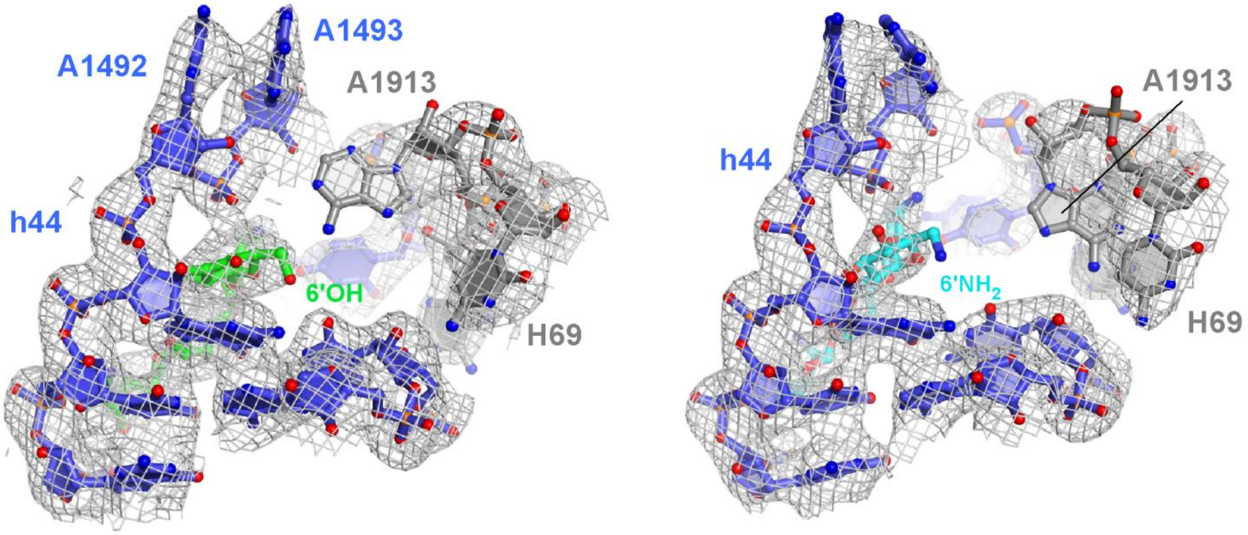

Supplementary Figure 12. Electron density of the decoding site in intermediate-rotated aminoglycoside-bound ribosomes. The decoding site region of the paromomycin- (left) and neomycin- (right) bound ribosome (intermediate-rotated) indicating the presence and absence of electron density for residue A1913 of H69, respectively. The 16S rRNA (blue), 23S rRNA (grey), h44-bound paromomycin (green) and h44-bound neomycin (light blue) are overlaid with feature-enhanced electron density maps[7], calculated in PHENIX and contoured at 1.4 standard deviations from the mean. The temperature factors for residue A1913 are as follows. Intermediate-rotated paromomycin-bound: ~120 $Å^2$; intermediate-rotated neomycin-bound: ~230 $Å^2$.

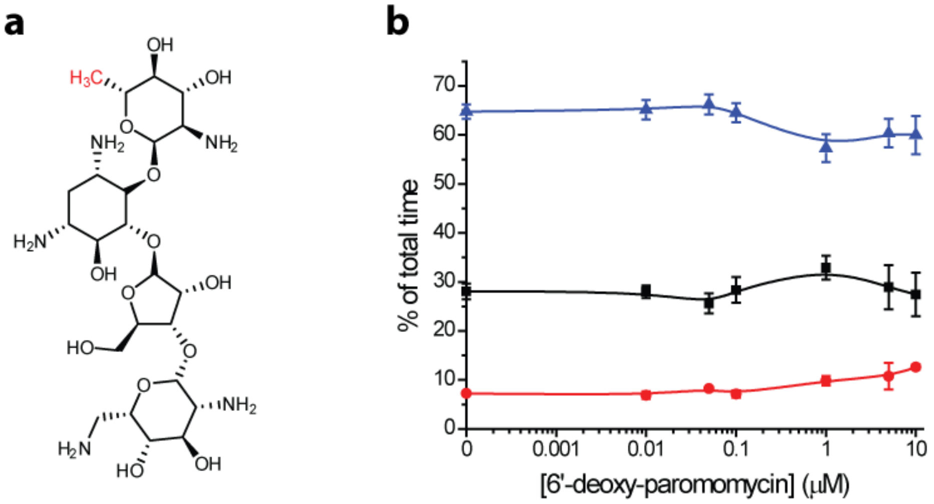

Supplementary Figure 13. The 6′ position of paromomycin ring I is critical for drug activity. (a) Chemical structure[8] of 6′-deoxy-paromomycin and (b) corresponding smFRET titration summary. Titrations are plotted as percent time spent in each FRET state calculated from the idealized data (**Methods**). Low-FRET (black); intermediate-FRET (red); high-FRET (blue). B-spline functions are fit to the data to aid in visualization only. Experiments were repeated on three separate days. The means ± standard deviations are plotted.

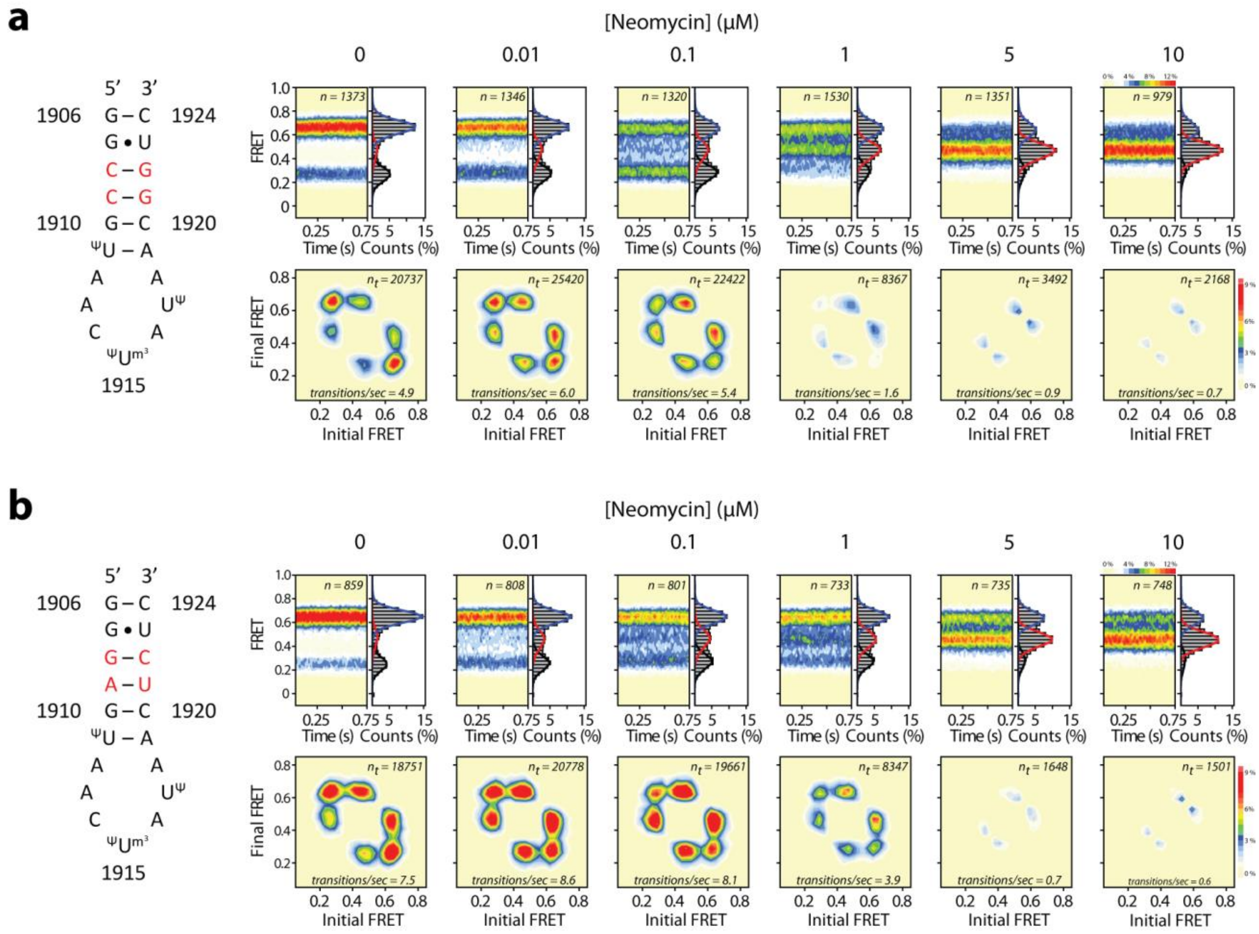

Supplementary Figure 14. Sequence mutations of the *E. coli* H69 to those found in humans alters intersubunit rotation dynamics and neomycin activity. (left) Secondary structure of the (a) wild-type *E. coli* H69 sequence and (b) the *E. coli* sequence altered (**Methods**) to contain four mutations (red) found in human 28S rRNA. (top panels) smFRET trajectories summed into FRET histograms reveal the population behaviors across a range of neomycin concentrations in (a) wild-type and (b) H69-mutant ribosomes. (bottom panels) Initial and final FRET values for each transition summed into two-dimensional histograms (transition density plots) show that transitions among distinct FRET states are markedly altered in the presence of neomycin. In the absence of drug, H69-mutant ribosomes exhibit notably faster intersubunit rotation dynamics than wild-type ribosomes. The H69 mutations shift the concentration of neomycin required to stabilize intermediate-FRET (compare wild-type 1 μM *vs*. H69 5 μM).

Supplementary Table 1: *in vitro* translation assays

	WT				A1408G			
	**IC_{50} (μM)**[a]	**SE (μM)**	***n***[b]	**SE**	**IC_{50} (μM)**	**SE (μM)**	***n***	**SE**
**Neomycin**	0.01447	0.0010	1.7	0.2	42	2.6	1.5	0.13
**Paromomycin**	0.0199	0.0016	1.6	0.2	1.6	0.087	1.4	0.090
**Ribostamycin**	0.126	0.0089	1.6	0.2	>300	N/A	N/A	N/A
**Neamine**	1.44	0.054	1.5	0.1	>300	N/A	N/A	N/A
**6'-*N*-acetyl-neomycin**	0.749	0.026	1.6	0.1	>300	N/A	N/A	N/A
**3-*N*-acetyl-neomcyin**	33.8	1.9	1.5	0.1	>300	N/A	N/A	N/A

[a] See **Methods** for calculations of the half-maximal inhibitory concentrations (IC_{50})
[b] *n* is the Hill coefficient (see **Methods**)

Supplementary References

1. Wang L*, et al.* Allosteric control of the ribosome by small-molecule antibiotics. *Nat Struct Mol Biol* **19**, 957-963 (2012).
2. Dunkle JA*, et al.* Structures of the bacterial ribosome in classical and hybrid states of tRNA binding. *Science* **332**, 981-984 (2011).
3. Feldman MB, Terry DS, Altman RB, Blanchard SC. Aminoglycoside activity observed on single pre-translocation ribosome complexes. *Nat Chem Biol* **6**, 54-62 (2010).
4. Barbieri CM, Pilch DS. Complete thermodynamic characterization of the multiple protonation equilibria of the aminoglycoside antibiotic paromomycin: a calorimetric and natural abundance 15N NMR study. *Biophys J* **90**, 1338-1349 (2006).
5. Kaul M, Barbieri CM, Kerrigan JE, Pilch DS. Coupling of drug protonation to the specific binding of aminoglycosides to the A site of 16 S rRNA: elucidation of the number of drug amino groups involved and their identities. *J Mol Biol* **326**, 1373-1387 (2003).
6. Kaul M, Pilch DS. Thermodynamics of aminoglycoside-rRNA recognition: The binding of neomycin-class aminoglycosides to the A site of 16S rRNA. *Biochemistry* **41**, 7695-7706 (2002).
7. Afonine PV*, et al.* FEM: feature-enhanced map. *Acta Crystallogr D Biol Crystallogr* **71**, 646-666 (2015).
8. Pathak R, Bottger EC, Vasella A. Design and synthesis of aminoglycoside antibiotics to selectively target 16S ribosomal RNA position 1408. *Helvetica Chimica Acta* **88**, 2967-2985 (2005).
